# Microplastic contamination of table salts from Taiwan, including a global review

**DOI:** 10.1038/s41598-019-46417-z

**Published:** 2019-07-12

**Authors:** Hyemi Lee, Alexander Kunz, Won Joon Shim, Bruno A. Walther

**Affiliations:** 1 Medipeace, #401, 30, Digital-ro 32gil, Guro-gu, Seoul 08390 Republic of Korea; 2 National Taiwan University, Department of Geosciences, No. 1, Sec. 4, Roosevelt Road, Taipei, 10617 Taiwan; 3 Oil & POPs Research Group, Korea Institute of Ocean Science and Technology (KIOST), Geoje, 53201 Republic of Korea; 4 Department of Biological Sciences, National Sun Yat-sen University, Gushan District, Kaohsiung City, 804 Taiwan

**Keywords:** Imaging, Imaging, Environmental impact, Environmental impact

## Abstract

Plastic pollution is a rapidly worsening environmental problem, especially in oceanic habitats. Environmental pollution with microplastic particles is also causing food consumed by humans to be increasingly polluted, including table salts. Therefore, we present the first study which focuses only on table salt products purchased in Taiwan which we examined for the presence of microplastics. We used Fourier transform infrared spectroscopy to identify the polymer type of each particle. Within 4.4 kg of salt, we detected 43 microplastic particles which averages to 9.77 microplastic particles/kg. The identified polymer types were, in descending abundance, polypropylene, polyethylene, polystyrene, polyester, polyetherimide, polyethylene terephthalate, and polyoxymethylene. We combined our novel results with those of previous studies to provide the first global review of microplastic contamination of table salts. We found that 94% of salt products tested worldwide contained microplastics, with 3 out of 27 polymer types (polyethylene terephthalate, polypropylene, polyethylene) accounting for the majority of all particles. Averaging over seven separate studies, table salts contain a mean of 140.2 microplastic particles/kg. With a mean annual salt consumption of ~3.75 kg/year, humans therefore annually ingest several hundred microplastic particles from salt alone.

## Introduction

Plastic pollution is a growing environmental problem in terrestrial habitats^1,2^ but even more so in coastal, riverine, and oceanic habitats^3–10^ including Taiwan^11–13^. Meanwhile, global plastic production and waste generation have been growing exponentially and reached about 335 million tons in 2016^14–16^. Of these, between 4.8 to 12.7 million metric tons are estimated to enter the oceans annually^17^.

Once in the environment, plastic objects and fragments (1) visually and structurally damage coastal, oceanic, and riverine environments, with negative effects on tourism, (2) spread invasive species, (3) damage and endanger ships, (4) cause injury and death of animals through ingestion and entanglement, and (5) eventually degrade to meso-, micro-, and nanoplastic particles which can enter the food chain directly or contaminate it via the leaching of their chemical and often toxic ingredients^2,9,18–33^. Meso-, micro-, and nanoplastic particles are generally defined to be the following size categories, respectively: <25 mm–5 mm, 5 mm–1 μm, and <1 μm^11^.

Potential impacts on human health are: (1) accidents; (2) the direct ingestion of microplastic particles (microplastics) via food and the possible resulting internal injury^33–41^; (3) the indirect contamination of air, food, and water with unhealthy substances leached from the plastics into the environment^2,42–46^; (4) and microplastics serving as pathogen vectors^32,47^.

Microplastics have already been found in various human foods: beer, drinking water, honey, seafoods, sugar^33^, and table salts^48–54^. Unlike rock salts which are mined underground and were formed in pre-modern times, human-consumed sea salts and lake salts are usually produced through a crystallization process whereby seawater or brine is evaporated by heat and wind. Therefore, pollutants found in these waters, including microplastics, find their way into the end product, namely table salt. Seven previous studies demonstrated the presence of microplastics in table salts which originated from 29 different countries^48–54^.

When we began this study in 2017, no study had examined table salts purchased in Taiwan. Therefore, we decided to examine the most commonly available salt products in Taiwan for microplastic contamination. The main purpose was to establish whether a potential health risk from microplastic ingestion was also present in products sold in Taiwan, and what the level of contamination was.

During the course of our study, several other similar studies came out, including Kim *et al*.^54^ which included four salt products purchased in Taiwan as part of their global study. While we thus lost the novelty of being the first to test products purchased in Taiwan, our study examined a higher number of products and thus remains the first detailed study focusing only on products purchased in Taiwan.

Given this recent accumulation of studies examining table salts from around the world, we also decided to conduct a global review of microplastic contamination of table salts in order to present the first global estimates of (1) the percentage of product samples which are contaminated, (2) the mean number of microplastics per kg of salt, (3) the estimated number of microplastics annually consumed by humans, (4) the sizes and shapes of microplastics, and (5) the number and percentages of identified polymer types. This novel and comprehensive review thus presents the first global estimates of microplastic contamination of table salts which are invaluable in order to be able to evaluate the potential health risks from the ingestion of microplastics via salt consumption.

## Results

### Numbers, shapes, sizes, and polymer types of microplastics

A total of 4.4 kg of salt (400 g * 11 products) was dissolved and filtered, and a total of 667 potential microplastic particles were visually detected on the filter papers (Table 1). All 667 particles were subjected to FTIR spectroscopy. For technical reasons, 20 of these particles did not return usable spectra and were only measured for size. Among the remaining 647 particles, 43 particles (6.6%) were identified to be plastic particles (Supplementary Fig. S1). The other 604 particles (93.4%) were identified mostly as natural materials, e.g., cotton, minerals, natural leaves, paper, or seeds. The 43 microplastic particles were categorized as 40 fragments (93%) and 3 fibers (7%) (Supplementary Appendix S1). There were 35, 7, and 1 particles of the size range of 1–500 μm, 500–1000 μm, and 1000–1500 μm, respectively. The mean, standard deviation, and range of the 43 particles were 342.3 ± 251.1 (minimum 89.7, maximum 1474.9) μm. The polymer types were 17 polypropylene (39.5%), 15 polyethylene (34.9%), 6 polystyrene (14.0%), 2 polyester (4.7%), 1 polyetherimide (2.3%), 1 polyethylene terephthalate (2.3%), and 1 polyoxymethylene (2.3%) particles.

**Table 1:** Number of particles identified as non-plastic or plastic in 11 salt products purchased in Taiwan. Column 2 gives the number of potential particles tested with FTIR spectroscopy.

Salt ID	No. tested	Only size	Non-plastic	Plastic particles			Total no. plastics	No. particles/kg
				Fibers	Fragments	Pellets		
1	38	2	35	1 (1 PET^a^)	0	0	1	2.5
2	30	0	28	0	2 (2 PP^aa^)	0	2	5.0
3	20	0	19	1 (1 PES^a^)	0	0	1	2.5
4	79	0	71	0	8 (4 PE^aaab^, 1 PEI^a^, 3 PP^aaa^)	0	8	20.0
5	23	0	21	0	2 (2 PP^aa^)	0	2	5.0
6	52	0	45	0	7 (3 PE^aab^, 4 PP^aaab^)	0	7	17.5
7	141	0	138	0	3 (1 PE^c^, 1 PES^a^, 1 PP^a^)	0	3	7.5
8	48	0	43	1 (1 PE^b^)	4 (3 PE^aab^, 1 PP^a^)	0	5	12.5
9	48	0	43	0	5 (5 PS^aaaab^)	0	5	12.5
10	131	12	115	0	4 (2 PE^aa^, 1 PAC^a^, 1 PS^a^)	0	4	10.0
11	57	6	46	0	5 (1 PE^b^, 4 PP^aaaa^)	0	5	12.5
Total	667	20	604	3	40	0	43	—

Column 3 gives the number of particles for which we only measured the particle’s size. Columns 4 and 5–8 give the number of non-plastic and plastic particles, respectively. For each of the plastic particles listed in columns 5–6, we give the polymer type (PE = polyethylene, PEI = polyetherimide, PES = polyester, PET = polyethylene terephthalate, PAC = polyacetal or polyoxymethylene, PP = polypropylene, PS = polystyrene) and its size (^a^1 μm ≤ particle <500 μm; ^b^500 μm ≤ particle <1000 μm; ^c^1000 μm ≤ particle <1500 μm); e.g., 3 PE^aab^ means 3 PE particles, 2 of size a and 1 of size b. Column 9 gives the number of microplastic particles/kg (calculated by dividing column 8 by 0.4 kg).

In most controls, <10 potential microplastic particles were found (Supplementary Table S1). None of the 121 potential microplastic particles (22 in water controls and 99 in air controls) were identified as plastic polymers using FTIR spectroscopy. Rather, most of these 121 particles were identified as natural fibers made of cotton or paper based on their spectra, but also their appearance. These results strongly suggest that environmental contamination with microplastics did not occur during our laboratory work, but that the microplastics identified by us (Table 1) originated from the salt products themselves.

We also determined the types of materials used for the packaging of the 11 salt products (Supplementary Table S1). The results demonstrate the variety of packaging materials used. The main materials were metal, paper, and polyethylene and polyethylene terephthalate, while polystyrene was used for plastic spoons inserted into the salt held inside the container.

### Global review

Our global review found eight relevant studies^48–55^ (Table 2). First, we reviewed the number of microplastic particles/kg detected by each study. For the global estimate, we only used the rows giving the combined numbers (see column 2 in Table 2). Yang *et al*.^48^ detected approximately 303 particles/kg which included both plastic and non-plastic particles. Of these particles, 84.9% were made of plastic, which yields a final estimate of approximately 303 * 0.849 = 257 microplastic particles/kg. Iñiguez *et al*.^50^ detected 128 particles/kg but only 93.3% of their particles were plastic particles, which yields a final estimate of 128 * 0.933 = 119 microplastic particles/kg (confirmed by Juan A. Conesa, in litt. 2018). Seth and Shriwastav^53^ detected 69.4 particles/kg but only ~80% of their particles were plastic particles, which yields a final estimate of 69.4 * 0.80 = 55.5 microplastic particles/kg (confirmed by A. Shriwastav, in litt. 2018). The other estimates were 1.88 microplastic particles/kg for Karami *et al*.^49^, 32.1 microplastic particles/kg for Gündoğdu^51^, 506 microplastic particles/kg for Kim *et al*.^54^, and 9.77 microplastic particles/kg for our study (Table 2). We excluded Kosuth *et al*.^55^ because they did not positively identify plastic particles but only “anthropogenic particles”. We also excluded Renzi and Blašković^52^ because they only used visual examination to determine whether a particle was plastic or not (Table 2). Because of the lack of spectroscopy analyses, the estimates in Renzi and Blašković^52^ may be overestimates “due to a technical limit of the experimental approach” (M. Renzi, in litt. 2018), particularly for particles <500 μm size^52^. Averaging over the seven included studies yields a mean ± standard deviation (and range) of 140.2 ± 183.9 (1.9–506.0) microplastic particles/kg.

**Table 2:** Number, sizes, and shapes of microplastic particles for our global review. Column 1 refers to: (1) Yang *et al*.^48^; (2) Karami *et al*.^49^; (3) Iñiguez *et al*.^50^; (4) Gündoğdu^51^; (5) Kosuth *et al*.^55^; (6) Renzi and Blašković^52^; (7) Seth and Shriwastav^53^ and A. Shriwastav in litt. (2018); (8) Kim *et al*.^54^; (9) our study. Column 2 refers to the salt types: SS = sea salt, LS = lake salt, RWS = rock/well salt, TS = table salt (source not described), combined = all salt types combined with the mean calculated across individual samples, not salt types. Column 3 gives the mean ± standard deviation (and the range) of the number of microplastic particles/kg. Column 4 gives the particles’ sizes: ^a^range, ^b^mean ± standard deviation, ^c^mean. Column 6 gives the identification method: FTIR = Fourier transform infrared spectroscopy, Raman = Raman spectroscopy.

ref	Salt type	No. particles/kg	Sizes (mm)	Shapes	Identification
1	5 SS	~616 ± 52 (550–681)	—	fibers, fragments, pellets, sheets	—
1	5 LS	~201 ± 130 (43–364)	—	fibers, fragments, pellets, sheets	—
1	5 RWS	~91 ± 86 (7–204)	—	fibers, fragments, pellets, sheets	—
1	combined	~303 ± 250 (7–681)	^a^0.045–4.3	fibers, fragments, pellets, sheets	FTIR of 152 particles (84.9% plastic)
2	14 SS	2.00 ± 3.26 (0–10)*	—	—	Raman of all particles
2	1 LS	1*	—	—	Raman of all particles
2	1 TS	1*	—	—	Raman of all particles
2	combined	1.88 ± 3.05 (0–10)*	^a^0.16–0.98, ^b^0.52 ± 0.17	fibers, films, fragments	Raman of all particles
3	16 SS	124.1 ± 57.4 (50–280)	—	fibers	—
3	5 RWS	139.0 ± 27.7 (115–185)	—	fibers	—
3	combined	127.6 ± 51.6 (50–280)	^a^0.03–3.5	fibers	FTIR of some^≠^ particles (93.3% plastic)
4	5 SS	46.0 ± 28.4 (16–84)	—	fibers, films, fragments	Raman of all particles
4	6 LS	37.5 ± 34.3 (8–102)	—	fibers, films, fragments	Raman of all particles
4	5 RWS	11.8 ± 2.8 (9–16)	—	fibers, films, fragments	Raman of all particles
4	combined	32.1 ± 28.7 (8–102)	^a^0.02–5, ^c^2.32	fibers, films, fragments	Raman of all particles
5	10 SS	205.8 ± 217.7 (46.7–806.0)^#^	—	—	analysis based on Rose Bengal stain
5	2 RWS	240.0 ± 179.6 (113.0–367.0)^#^	—	—	analysis based on Rose Bengal stain
5	combined	211.5 ± 204.6 (46.7–806.0)^#^	^a^0.1–5, ^c^1.09	fibers, fragments	analysis based on Rose Bengal stain
6	11 SS	7882 ± 8861 (20–19820)	^a^0.004–4.6	fibers, foams, fragments, pellets, sheets	visual identification only
7	8 SS	69.38 ± 17.3 (56.0–103.0)	^a^0.025–7.0	fibers, fragments	FTIR of ~1.5% particles (~80% plastic)
8	28 SS	675 ± 2560 (0–13629)	^—^	^—^	FTIR of most^≠^ particles
8	2 LS	245 ± 307 (28–462)	^—^	^—^	FTIR of all particles
8	9 RWS	38 ± 55 (0–148)	^—^	^—^	FTIR of all particles
8	combined	506 ± unknown^§^ (0–13629)	^a^0.1–5.0	fibers, fragments, pellets, sheets	FTIR of most^≠^ particles (91% plastic)
9	10 SS	9.50 ± 6.10 (2.50–20.00)	^a^0.09–1.47, ^b^0.34 ± 0.26	fibers, fragments	FTIR of all particles
9	1 RWS	12.50	^a^0.15–0.71, ^b^0.33 ± 0.23	fragments	FTIR of all particles
9	combined	9.77 ± 5.86 (2.50–20.00)	^a^0.09–1.47, ^b^0.34 ± 0.25	fibers, fragments	FTIR of all particles (6.6% plastic)

~Numbers were approximated from Figure 2 in Yang *et al*.^48^ and are therefore only our best approximations.

*Iñiguez *et al*.^50,58^ pointed out that Karami *et al*.^49^ used a filter with a pore size of 149 μm which meant that any particles smaller than the pore size were able to pass through the filter’s pores.

^#^Numbers refer to “anthropogenic particles” which Kosuth *et al*.^55^ assumed were synthetic particles (but may not all be plastic particles) based on their identification method of using a Rose Bengal stain.

^§^Kim *et al*.^54^ did not report an overall standard deviation.

^≠^Exact number was not given.

Second, we reviewed sizes and shapes. Again excluding Kosuth *et al*.^55^ and Renzi and Blašković^52^, mean sizes were only reported in three out of the seven studies (Table 2). Averaging over these three studies yields a mean ± standard deviation (and range) size of 1.06 ± 1.10 (0.34–2.32) mm. Size ranges were reported in all eight studies, and the smallest and largest size were 0.004 mm and 7.00 mm, respectively (Table 2). All studies identified fibers, eight out of nine studies identified fragments (Iñiguez *et al*.^50^ being the only exception), and films, foams, pellets, and sheets were also identified.

Third, we reviewed the mean number of polymer types which was 8.1 types across the seven included studies (Table 3). In total, 27 polymer types were detected, but only two polymer types (namely polyethylene terephthalate and polyethylene) were detected in all seven studies while polypropylene was detected in six out of seven studies. These three polymer types also have the highest mean percentages, accounting for 63.7% of records. The remaining 24 polymer types were only detected by one to four studies and all had mean percentages of <7.0%. The mean percentage of polymer types across studies and the number of studies which detected that polymer type correlate significantly (columns 10–11 in Table 3; Spearman rank correlation, n = 21, Z = 3.61, rho = 0.81, p = 0.0003).

**Table 3:** Polymer types of microplastics for our global review. Column 1 lists polymer types in descending order of their overall mean percentage across six studies (column 10). It is possible that some of the types are synonyms (e.g., polymethyl-methacrylate, poly methyl acrylate, polyacrylate). Column 2 lists the abbreviation of each polymer type used in the respective studies (^$^note that the abbreviation used for polyoxymethylene is often POM, but POM clashed with the abbreviation for polymerized, oxidized material used by Yang *et al*.^48^). Columns 3–9 list the percentage or presence of polymer types identified by (ref.^1^) Yang *et al*.^48^; (ref.^2^) Karami *et al*.^49^; (ref.^3^) Iñiguez *et al*.^50^; (ref.^4^) Gündoğdu^51^; (ref.^5^) Table 2 in Seth and Shriwastav^53^; (ref.^6^) Kim *et al*.^54^ reported no global means but noted that the four most common polymers were PET, PE, PP, and teflon; therefore, X denotes presence; (ref.^7^) our study. Column 10 gives the mean percentage calculated across columns 3–7 and 9, and column 11 gives the number of studies among the seven studies which identified the respective polymer type.

Polymer type	Abbreviation	ref.^1^	ref.^2^	ref.^3^	ref.^4^	ref.^5^	ref.^6^	ref.^7^	Mean %	No. of studies
polyethylene terephthalate	PET	16.3	6.7	89.3	14.5	23.5	X	2.3	25.4	7
polyethylene	PE	8.5	33.3	3.5	22.9	16.0	X	34.9	19.9	7
polypropylene	PP	4.7	40.0	7.2	19.2	0.0	X	39.5	18.4	6
cellophane	CP	39.5	0.0	0.0	0.0	0.0	0.0	0.0	6.6	1
polyisoprene/polystyrene	PS	0.0	6.7	0.0	0.0	17.7	X	14.0	6.4	4
polyester	PES	7.0	0.0	0.0	0.0	23.3	0.0	4.7	5.8	3
polyamide-6	nylon-6, NY6, PA-6	0.0	3.3	0.0	8.7	19.4	X	0.0	5.2	4
polyurethane	PU	0.0	0.0	0.0	17.5	0.0	X	0.0	2.9	2
polyvinylchloride	PVC	0.8	0.0	0.0	11.6	0.0	X	0.0	2.1	3
polyacrylonitrile	PAN	1.6	10.0	0.0	0.0	0.0	0.0	0.0	1.9	2
poly(1-butene)	PB	8.5	0.0	0.0	0.0	0.0	0.0	0.0	1.4	1
polymethyl-methacrylate	PMMA	0.0	0.0	0.0	5.7	0.0	0.0	0.0	0.9	1
polymerized, oxidized material	POM	4.7	0.0	0.0	0.0	0.0	0.0	0.0	0.8	1
polyalkene	PAK	3.1	0.0	0.0	0.0	0.0	0.0	0.0	0.5	1
PE and PP copolymer	PE-PP	2.3	0.0	0.0	0.0	0.0	0.0	0.0	0.4	1
polyacetal/polyoxymethylene	PAC^$^	0.0	0.0	0.0	0.0	0.0	0.0	2.3	0.4	1
polyetherimide	PEI	0.0	0.0	0.0	0.0	0.0	0.0	2.3	0.4	1
ethylene vinyl acetate	EVA	0.8	0.0	0.0	0.0	0.0	X	0.0	0.1	2
cellulose	CL	0.8	0.0	0.0	0.0	0.0	0.0	0.0	0.1	1
poly methyl acrylate	PMA	0.8	0.0	0.0	0.0	0.0	0.0	0.0	0.1	1
poly(vinyl acetate:ethylene)	—	0.8	0.0	0.0	0.0	0.0	0.0	0.0	0.1	1
acrylic	—	0.0	0.0	0.0	0.0	0.0	X	0.0	—	1
paraffin wax	PW	0.0	0.0	0.0	0.0	0.0	X	0.0	—	1
phenoxy resin	PR	0.0	0.0	0.0	0.0	0.0	X	0.0	—	1
polyacrylate	PA	0.0	0.0	0.0	0.0	0.0	X	0.0	—	1
polycarbonate	PC	0.0	0.0	0.0	0.0	0.0	X	0.0	—	1
teflon	—	0.0	0.0	0.0	0.0	0.0	X	0.0	—	1
Total no. of polymers detected	27	15	6	3	7	5	14	7	—	-

## Discussion

The identification by FTIR spectroscopy is one of the most widely accepted methods to confirm the polymer types of microplastics^56,57^. Using this method, we found 43 microplastics within 4.4 kg of salt from 11 salt products, or 9.77 microplastic particles/kg (Table 2). Assuming that our 20 unidentified particles had the same percentage of microplastic particles as the 647 identified particles (namely 6.6%, cf. Table 1), these 20 particles would add only 20 * 0.066 = 1.33 microplastic particles to the total of 43 identified particles. Such a correction yields a slightly higher estimate of 10.08 microplastic particles/kg. Therefore, the possible error because of the 20 unidentified particles is about 3.1%.

The 43 microplastics were mostly fragments, with only three of them being fibers, and had a mean size of 342.3 μm. We detected seven different polymer types, with the most common by far being polypropylene and polyethylene. In our two types of controls, we found no microplastics which strongly suggests that environmental contamination during laboratory work did not occur.

Our results fit well with previously published results which we summarized in a global review (Tables 2,3). Although our estimate of microplastic particles/kg is relatively low, it is within the range of previously published results. While our mean size is the lowest of three studies, it is also well within the range of published results, as are the shapes which we detected. Finally, the two most commonly detected polymer types in our study were among the three most commonly detected polymer types across all studies. We also detected the most commonly detected polymer type, namely polyethylene terephthalate, in our study, although at the lowest rate across the six studies. So far, a total of 27 polymer types have been detected in salt products, but this number will surely increase because of the large number of polymer types which continuously enter the environment^16,17^. We also found a significant positive correlation between the mean percentage of polymer types across studies and the number of studies which detected that polymer type. In other words, the most common polymer types are found in most studies and also make up most of the detected particles, which is a result to be expected.

Our global review supports the supposition that almost all salt products are now contaminated with at least some microplastics. Our study as well as four other studies^48,50,51,53^ found microplastics in every examined salt product (71 out of 71, cf. Table 2), while Kim *et al*.^54^ detected microplastics in 36 out of 39 examined salt products, and Karami *et al*.^49^ detected microplastics in 11 out of 16 examined salt products. Karami *et al*.^49^ had by far the lowest detection rate of all studies which may well be due to their choice of filter size^50,58^. However, even including Karami *et al*.’s^49^ results, 118 out of 126 salt products (94%) tested worldwide contained microplastics. Such a level of contamination is rather troublesome given that one could be justified to demand that no salt product should be contaminated.

The variation in microplastic particles/kg across the seven studies is surprising as it covers more than two magnitudes from the lowest by Karami *et al*.^49^ to the highest by Kim *et al*.^54^ (Table 2; Renzi and Blašković’s^52^ mean is even higher, but we excluded it for reasons mentioned above). This large amount of variation is also apparent when we mapped the global distribution of the mean number of microplastic particles/kg (Supplementary Fig. S2, see also Figure 2 in Kim *et al*.^54^). We assume that both the variation in the actual contamination of the products (e.g., driven by plastic contamination of rivers and seawaters^54^) and the variation in the laboratory procedures used in the seven studies explains this rather large variation. We therefore suggest that standard protocols should be agreed upon (e.g., ref.^39^) and that, at a minimum, researchers should report all the different data types presented in Tables 2,3. Given that the seven studies used either FTIR or Raman spectroscopy, we can exclude another possibility, namely the misidentification of natural fibers as plastic fibers^59^.

While some of the contamination of the salt products happens during the processing and packaging stages (see Discussion below), it is likely that most contamination is due to environmental contamination. We found some new evidence to support this supposition of environmental contamination being the main culprit. Hidalgo-Ruz *et al*.^60^ in their Table 7 list 13 polymer types which were detected in 42 studies which identified microplastics recovered from the marine environment, with polyethylene, polypropylene, and polystyrene detected far more often than the other 10 polymer types. Interestingly, the number of studies in which each respective polymer type was detected in Hidalgo-Ruz *et al*.’s^60^ review correlates significantly with the number of studies in which the respective polymer type was detected in our global review (column 11 in our Table 3; Spearman rank correlation, n = 28, Z = 3.38, rho = 0.65, p = 0.0007). Thus, the plastic polymers most commonly detected in the marine environment are generally also the plastic polymers most commonly found in table salts (see also Kim *et al*.’s^54^ results).

Further evidence for environmental contamination comes from comparisons of salt types. Yang *et al*.^48^, Gündoğdu^51^, Kim *et al*.^54^, and, to a lesser degree, Karami *et al*.^49^ and our study found that the number of microplastics generally followed a trend of sea salts > lake salts > rock/well salts (Table 2). This trend is to be expected given that oceans are likely more polluted than lakes, and that rock and well salts should actually be unpolluted. However, this trend was not supported by the results in Iñiguez *et al*.^50^ and Kosuth *et al*.^55^.

The contamination of practically all salt products, even those which originate from what should be uncontaminated rock and well sources, suggests that some (although relatively minor) contamination does not originate from the salts but from sources during the processing and packaging of the products^48–50,52–54^. We can add some further evidence to this supposition. The five microplastic particles of salt 9 are all polystyrene (Table 1) and also all have a bluish colour (Supplementary Appendix S1), which could perhaps be tiny fragments from the blue spoon made of polystyrene and provided inside the salt product (Supplementary Table S1). The one polystyrene microplastic particle in salt 10 (Table 1) has a pinkish colour (Supplementary Appendix S1), while the provided spoon also made of polystyrene was red (Supplementary Table S1). Therefore, this polystyrene particle might also originate from the spoon. The only other matches between the packaging and the detected microplastics are for the polyethylene packaging used for salts 7 and 10, in which we also detected polyethylene particles. While this is only anecdotal evidence, it suggests that packaging, in this case the spoons provided in the salt product, could also be sources of microplastic contamination. Future studies should investigate in detail whether packaging, including plastic spoons, add microplastic contamination to food products.

Our study, like other studies, found many other particles of unknown composition in addition to the microplastics. Most of the other studies did not identify these other particles but Karami *et al*.^49^ identified several pigments, and Kim *et al*.^54^ identified 2.5% natural polymers and 0.7% mineral particles, while we identified many natural materials such as cotton, minerals, natural leaves, paper, or seeds. We did not dissolve natural materials before filtration (see Methods), as most other studies did, and this different methodology may explain our relatively low percentage of microplastic particles among all potential particles (cf. column 6 in Table 2). To conclude, these non-plastic particles in table salts can just be mineral or organic impurities, or they could be potentially harmful particles, such as pigments.

Our global review also enabled us to calculate estimates of the annual consumption of microplastics. Salt consumption is about 11.3 g/day for men and 8.8 g/day for women aged 19-64 years in Taiwan^61^, thus averaging about 3.67 kg/year. For the 11 salt products consumed in Taiwan, we detected 9.77 microplastic particles/kg, which yields an annual intake of 35.8 microplastic particles/year. According to the new guidelines of the World Health Organization^62^, adults should consume ≤5 g/day which yields a maximum input of 1.825 kg/year. Our global review estimated 140.2 microplastic particles/kg which would result in an annual intake of 255.9 microplastic particles/year. However, the global daily salt consumption in 2010 was actually estimated to be about 9.88–10.2 g/day^63^; taking the midpoint, this amounts to 3.66 kg/year and an annual intake of 513.8 microplastic particles/year. In 2016, the WHO Media Centre^64^ estimated global daily salt consumption to be about 9.0–12.0 g/day; taking the midpoint, this amounts to 3.83 kg/year and an annual intake of 537.4 microplastic particles/year. In summary, the best available current evidence suggests that, from salt alone, humans on average ingest several hundred microplastic particles per year. Given that one may be justified to demand that there should be no microplastics in table salts, any contamination should be of concern. However, it should also be noted that these numbers are far below estimates of about 11000 microplastic particles/year for the consumption of mollusks^35^. With the current exponential increase in plastic use, we can expect such contamination levels to increase everywhere, including sea and lake salts.

A growing number of reports have addressed the possible human health effects of the consumption of microplastics^1,2,32,33,40,41,45,65–67^. Three possible impacts have been described: direct ingestion of microplastics and the possible internal injury; the indirect contamination of air, food, and water; and the possibility of microplastics serving as pathogen vectors. However, whether these possible impacts translate into actual significant health risks is still largely unknown. As GESAMP^65^ stated, “the potential ecological and human health risks of microplastics are relatively new areas of research, and there is currently a large degree of uncertainty surrounding this issue,” which is an opinion also reflected by Miller *et al*.’s^41^ review. Meanwhile, the EFSA Panel on Contaminants in the Food Chain^40^ concluded that the presence of microplastics in seafood probably has a small effect on people’s overall exposure to additives or contaminants.

## Conclusions

This is the first study which exclusively examined salt products from Taiwan for microplastics, and it is also the first study to include a comprehensive global review of similar studies. All the 11 salt products from Taiwanese markets contained microplastics, and 94% of salt products tested worldwide contained microplastics. Such contamination results in an estimated mean annual consumption of several hundred microplastic particles. As pointed out above, there is no consensus yet whether significant health risks exist and what quantity of ingestion of microplastics might be considered harmful or not. However, increasing amounts of plastic are entering into various habitats, whereby this plastic pollution impacts ocean ecosystems the most. Clearly, seafoods and sea salts are contaminated with microplastics, and until we know more about the potential health effects, this fact remains worrying, especially given that this impact will increase in the coming decades. Therefore, research about the presence of microplastics in food products must be continued, but also much more research is required to establish the severity of the impact on human health.

## Methods

### Purchase of edible table salts from Taiwanese markets

Taiwan is an East Asian nation consisting of one large island (named Taiwan Island) and several much smaller islands and island groups and has a human population of approximately 23.5 million people. It has the 22^nd^ highest GDP per nation and the 30^th^ highest GDP per capita income in the world, with people consuming much local food, but also food imported from all over the world.

Over three weeks in June 2017, we visited 28 different supermarkets in Taipei, Taiwan, and wrote down every edible table salt product which was for sale. We then purchased the 10 most commonly sold sea salt products (i.e., the products which made the top of our list when adding up their presence in the 28 supermarkets). We also added one rock salt product for comparison. We assume that these 11 products are all regularly and widely consumed by Taiwanese people. The commercial names of these products cannot be made public for privacy reasons.

For each product, we obtained information for packaging, weight per package, origin of salt, and salt type from the original package or the company’s website given on the package (in most cases translated from Chinese) (Supplementary Table S1). The 11 products were produced by nine different companies whereby salts 1–3 were produced by the same company; therefore, these three products could be from the same source (Tainan, Taiwan), and thus they may just be the same salt packaged in three different ways. Given the information on the packaging, we however assume that the other salts 4–11 all come from different sources.

### Extraction of non-salt particles

We conducted all laboratory work at the Department of Geosciences at National Taiwan University between July 2017 and January 2018.

For all laboratory procedures, we produced particle-free water as follows^48,49^. Whenever needed, we filtered several liters of tab water through a 5μm pore size filter paper made by Advantec. The filtering system consisted of a vacuum pump connected via a rubber tube to a collecting flask. The filtered water was stored in a metal tank and was used exclusively for all further laboratory work. Before the next steps, all equipment parts were first cleaned twice with filtered water, after which the tops of the funnel and all flasks were covered with aluminium caps^48,49^. The caps were only removed when needed.

To dissolve the salt, we transferred the salt directly from its package to a 1-liter glass flask using a metal spoon. The weight of the salt was measured with a Precisa XS 6250D electronic scale on which the flask had been placed. After 100 g of salt had been transferred to the flask, 800 ml of filtered water were added, and the flask’s open top was then immediately covered with a cap. The combination of 100 g of salt and 800 ml of water had been established as the best solution to dissolve all the salt in preliminary tests. We then shook the flask manually to accelerate the salt’s dissolution, but it could nevertheless take up to 30–60 minutes for all the salt to become visibly dissolved.

To filtrate the salty solution, we used the same setup which was used to make filtered water described above. After removing the cap, we immediately poured the salty solution into the funnel upon which it was sucked through a new 5 μm pore size filter paper which would take less than one minute. The funnel was then further rinsed with filtered water to suck any residue remaining in the funnel through the filter. Thereafter, we immediately placed the filter paper into a new petri dish, covered it with a cover, and sealed it with tape. The filter paper in each petri dish was dried at room temperature for one night.

We repeated this process three more times, each time with a new flask, filter paper, and petri dish until we had dissolved 400 g of each salt product.

Besides using filtered water and caps, we further minimized contamination of samples by always wearing a laboratory suit which does not shed fibers^49^. Concurrently with the laboratory work described above, we always conducted two controls:

1. Control water: We filled one flask with 800 ml of filtered water and sucked the water through a new filter paper to check for contamination in the water and/or the filter paper^49^.

2. Control air: We poured 800 ml of filtered water into an open wide mouth bowl and placed it directly next to the laboratory equipment so that the water could catch airborne particles. At the end of each laboratory run of dissolving and filtering one of the 11 salt products, we then also filtered the water from the bowl through a new filter paper.

### Visual count of non-salt particles

We examined each filter paper using a Hamlet NSZ-606 Zoom Stereo Microscope (range of magnification 0.8x–5x). We counted the number of potential microplastic particles which we could visibly detect for each filter paper and categorized them into three main groups based on overall shape using the definitions established by Karami *et al*.^49^: fibers/filaments (thin, straight and often cylindrical particles), fragments (jagged and irregular shaped particles which often have an uneven surface), and pellets (rounded particles). All particles were photographed.

### Fourier transform infrared (FTIR) spectroscopy

We analyzed 667 potential microplastic particles with our FTIR spectroscopy setup described below. Of these particles, 647 particles were analyzed with FTIR spectroscopy while the remaining 20 particles were only measured for size because they did not return usable spectra. In January, February, and June 2018, we used an FTIR microscope (Nicolet iN 10 Infrared Microscope, Thermo Scientific, Waltham, Massachusetts, USA) in attenuated total reflectance (ATR) mode, equipped with a liquid nitrogen-cooled mercury cadmium telluride (MCT) detector at the Korean Institute of Ocean Science and Technology for our spectroscopy measurements. We used a diamond tip, and the spectra were recorded as 64 scans in the spectral range of 650–4000 cm^−1^.

Plastic polymer types were identified by matching the sample’s IR spectrum with the spectra stored in an FTIR polymer spectrum library which is already integrated into the measuring program of the FTIR microscope^68^. To measure the size of each particle, a ruler built into the measuring program of the FTIR microscope was adjusted to measure the maximum dimension of the particle. This equipment has been used for various studies of microplastics before (e.g., refs^5,56^).

The results of our spectroscopy analyses are available in the Supplementary Appendix S1.

### Global review

In October 2018, we used Google Scholar and Web of Science to obtain all previous studies which investigated the presence of microplastic particles in salt products. We used combinations of obvious keywords (contamination, microplastic, plastic pollution, salt, table salt) to find studies whose title and abstract we then checked for relevance. For each of the eight studies^48–55^ which we found, we also checked (1) the references cited within that study, and (2) the references which cited that study (both in Google Scholar and Web of Science). To add missing information to our global review, we also contacted the corresponding authors of each study to obtain additional information, with several replying (see Acknowledgements).

### Statistical analyses

Yang *et al*.^48^, Gündoğdu^51^, and Seth and Shriwastav^53^ used a one-way analysis of variance (ANOVA) to test for mean differences of the abundance of microplastics among salt types or products while Kim *et al*.^54^ used a Mann-Whitney U-test. However, we *a priori* refrained from doing so because of (1) our small sample sizes for one of the two salt types (only one rock salt product) and (1) the small sample sizes of plastic particles which we detected in each product (1–8 particles, Table 1). The remaining four studies testing salts for microplastics^49,50,52,55^ also refrained from the use of any statistical tests. Instead, we used standard descriptive statistics, such as the mean, standard deviation, range, and percentage, to describe the results of our laboratory work. For the global review, we used the non-parametric Spearman rank correlation because the data were not normally distributed.

## Supplementary information

Microplastic contamination of table salts from Taiwan, including a global reviewPlastic pollution is a rapidly worsening environmental problem, especially in oceanic habitats. Environmental pollution
