# Supplementary material for: Microplastic contamination of table salts from Taiwan, including a global review
Source: Sci Rep. 2019 Jul 12;9:10145. doi: 10.1038/s41598-019-46417-z (PMC6626012; doi:10.1038/s41598-019-46417-z)

Supplementary Information (one Appendix, one Table, two Figures) for:

Microplastic contamination of table salts from Taiwan, including a global review

Hyemi Lee[1], Alexander Kunz[2], Won Joon Shim[3], & Bruno A. Walther[4]

3 July 2019

[1]Medipeace, #401, 30, Digital-ro 32gil, Guro-gu, Seoul, 08390, Republic of Korea
[2]National Taiwan University, Department of Geosciences, No.1, Sec. 4, Roosevelt Road, Taipei 10617, Taiwan (R.O.C.)
[3]Oil & POPs Research Group, Korea Institute of Ocean Science and Technology (KIOST), Geoje 53201, Republic of Korea
[4]Department of Biological Sciences, National Sun Yat-sen University, Gushan District, Kaohsiung City, 804, Taiwan (R.O.C.)

Appendix S1

Photos of the 43 microplastic particles summarized in Table 1. For each particle, we give the shape, size, and plastic polymer type (for abbreviations, see Table 1).

Salt 1

Particle 1. Fiber, 226.18 μm, PET.

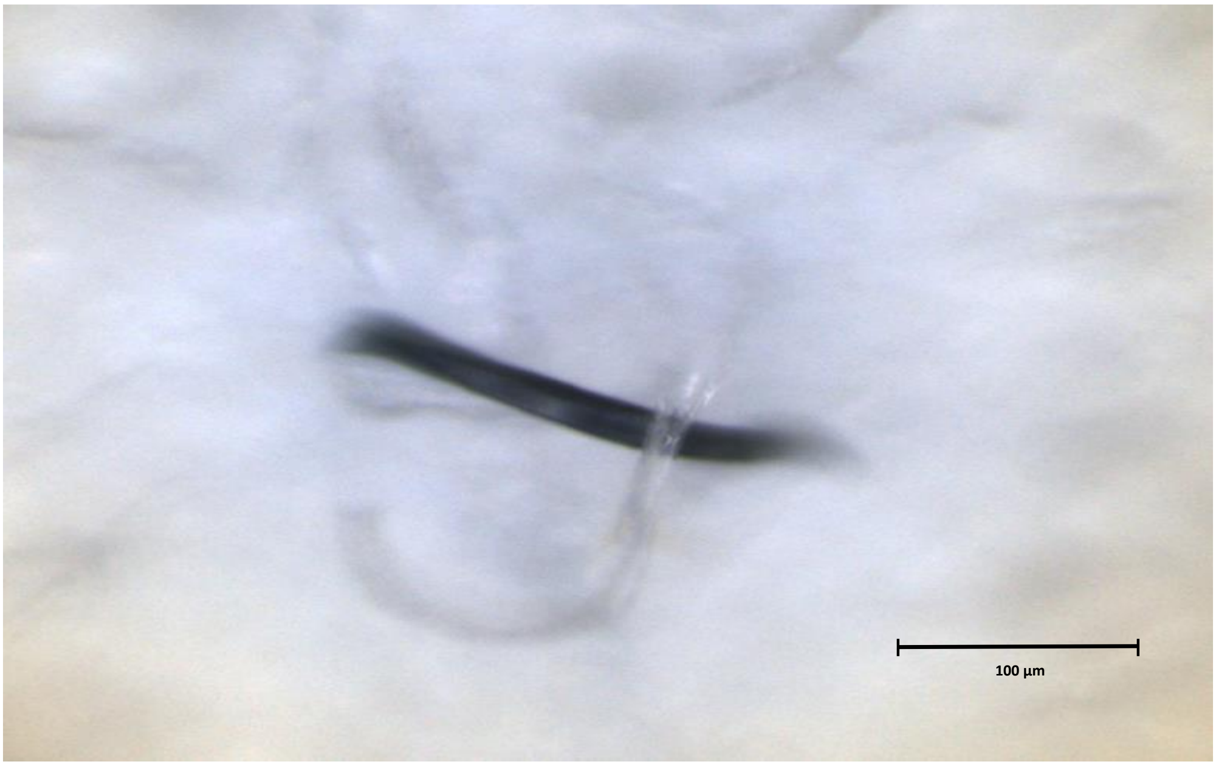

Salt 2

Particle 2. Fragment, 249.12µm, PP.

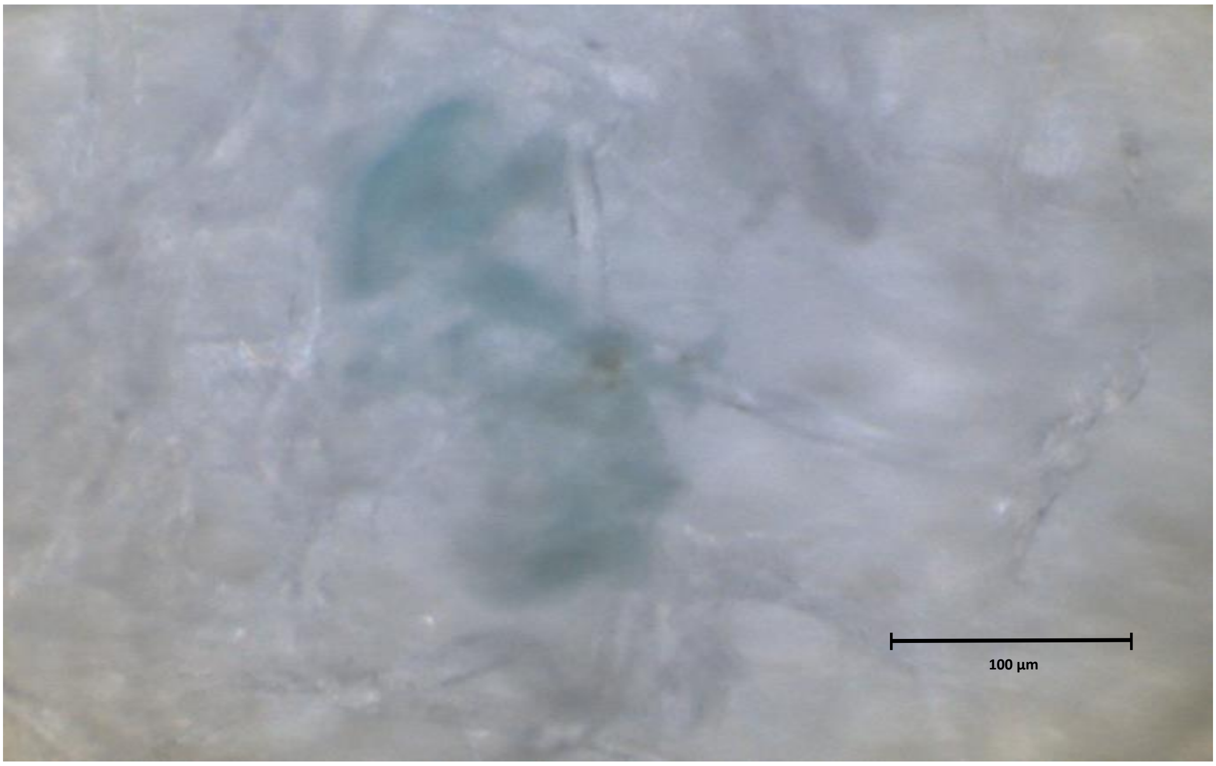

Particle 3. Fragment, 123.64 µm, PP.

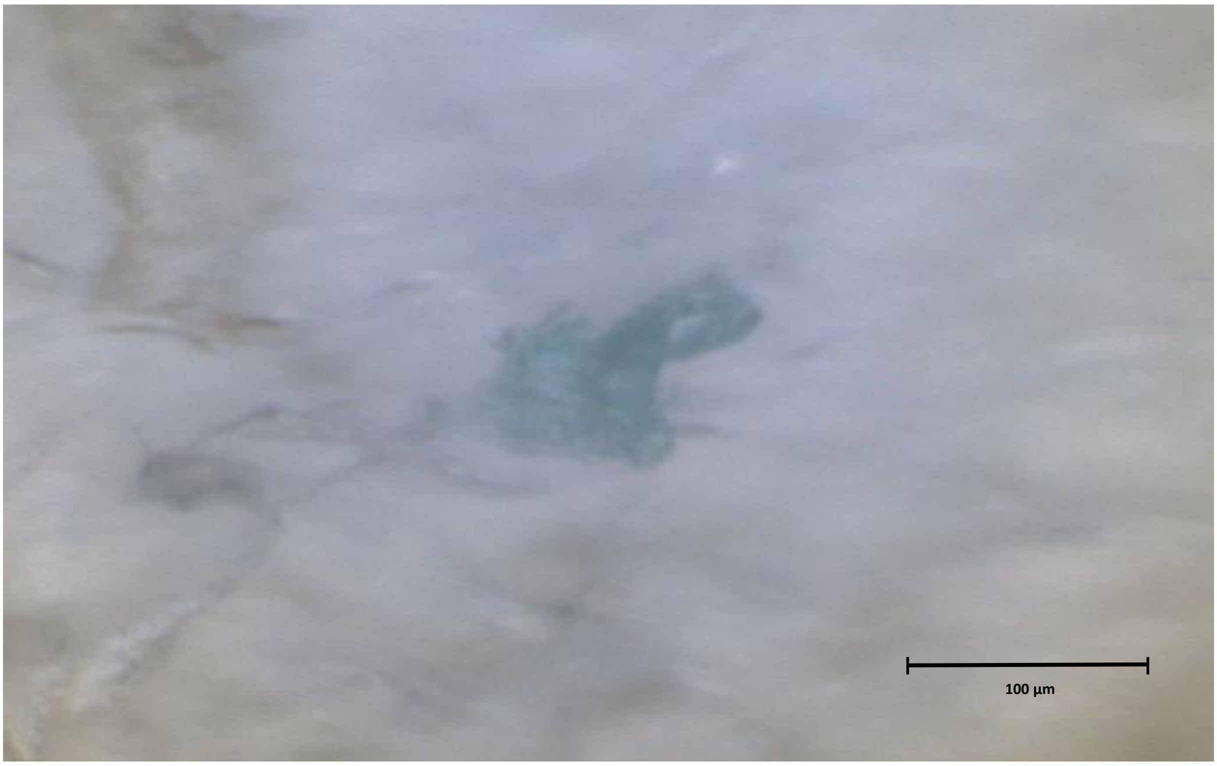

Salt 3

Particle 4. Fiber, 449.46 μm, PES.

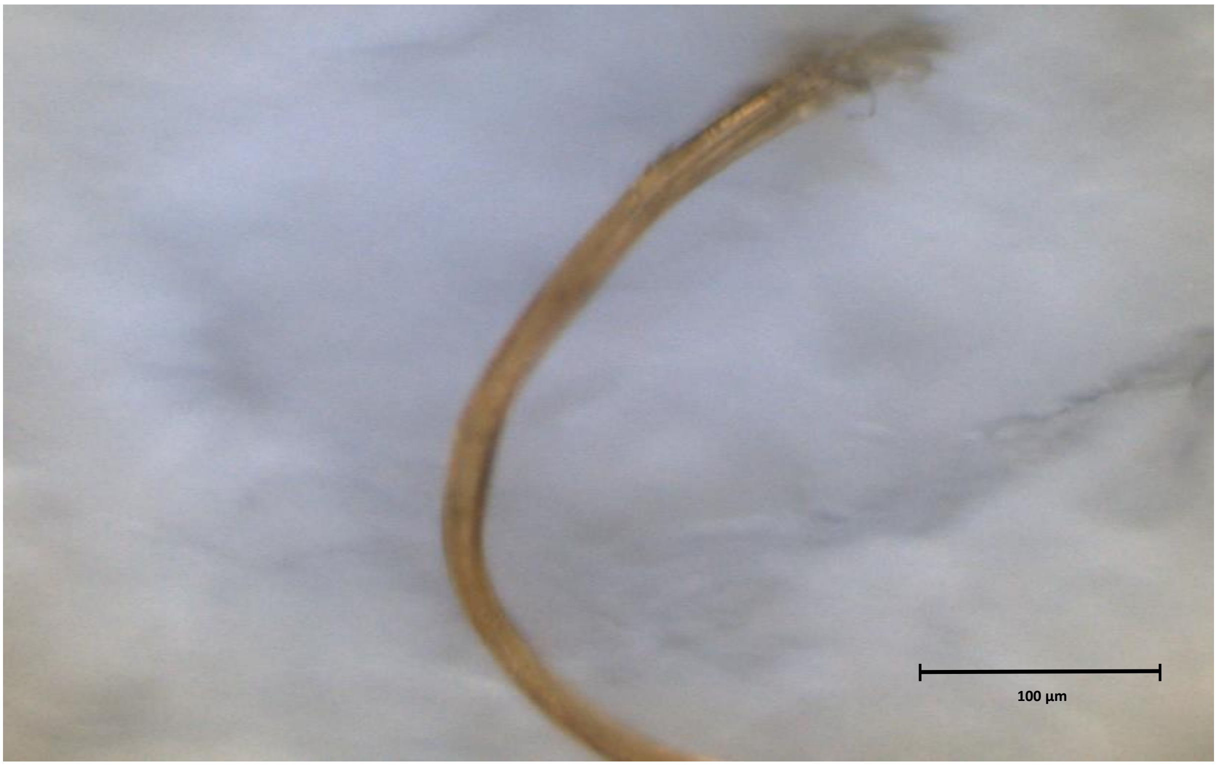

Salt 4

Particle 5. Fragment, 218.66 µm, PEI.

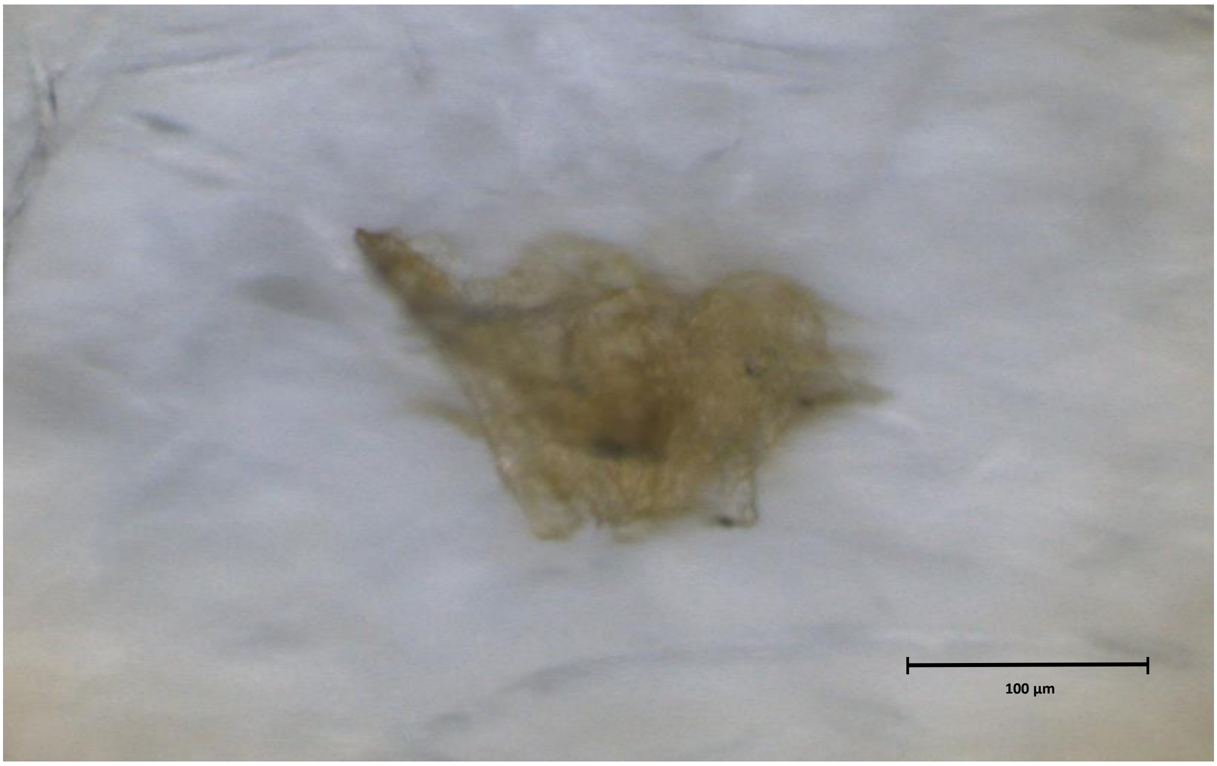

Particle 6. Fragment, 183.31 µm, PE.

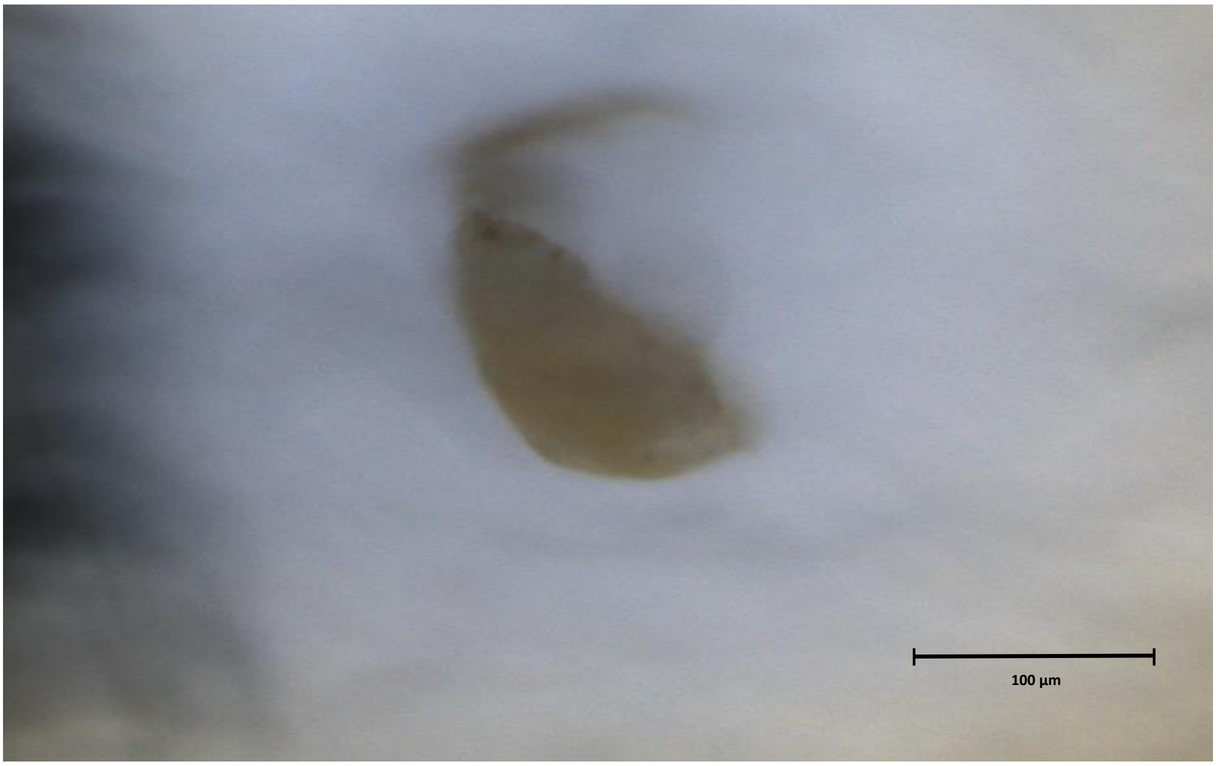

Particle 7. Fragment, 150.38 µm, PP.

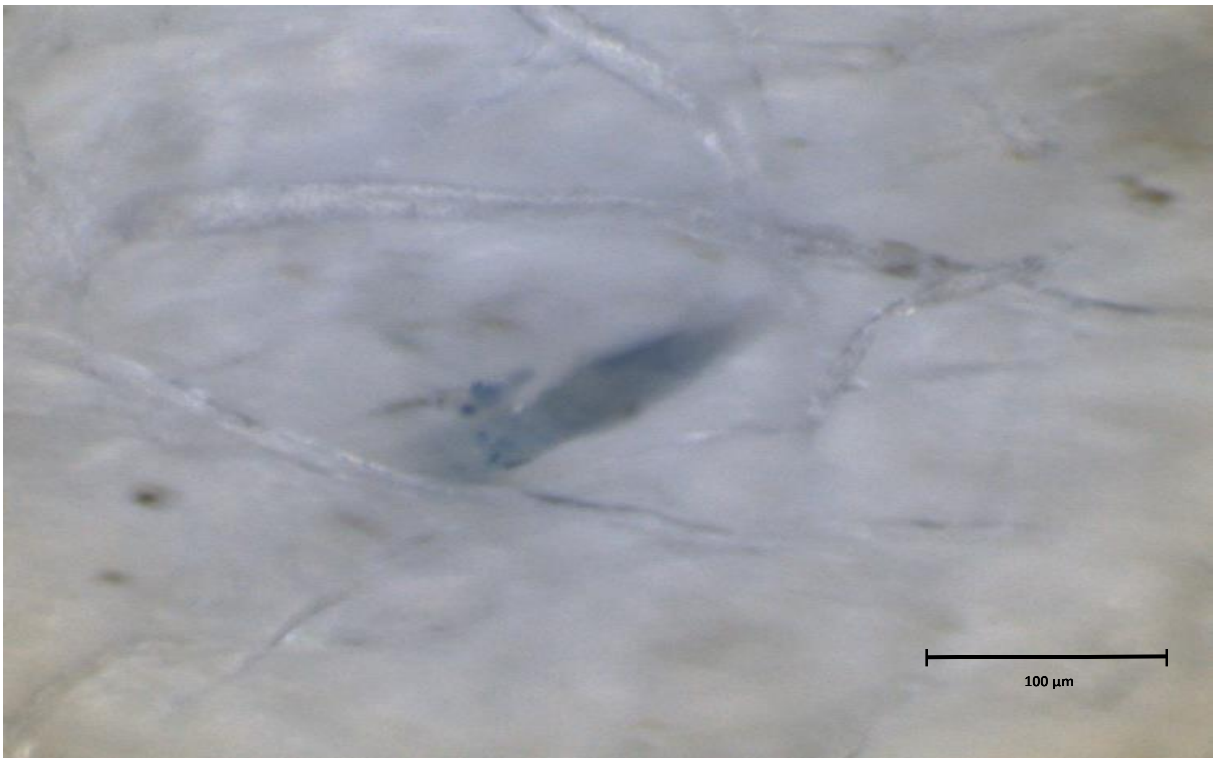

Particle 8. Fragment, 277.51 µm, PE.

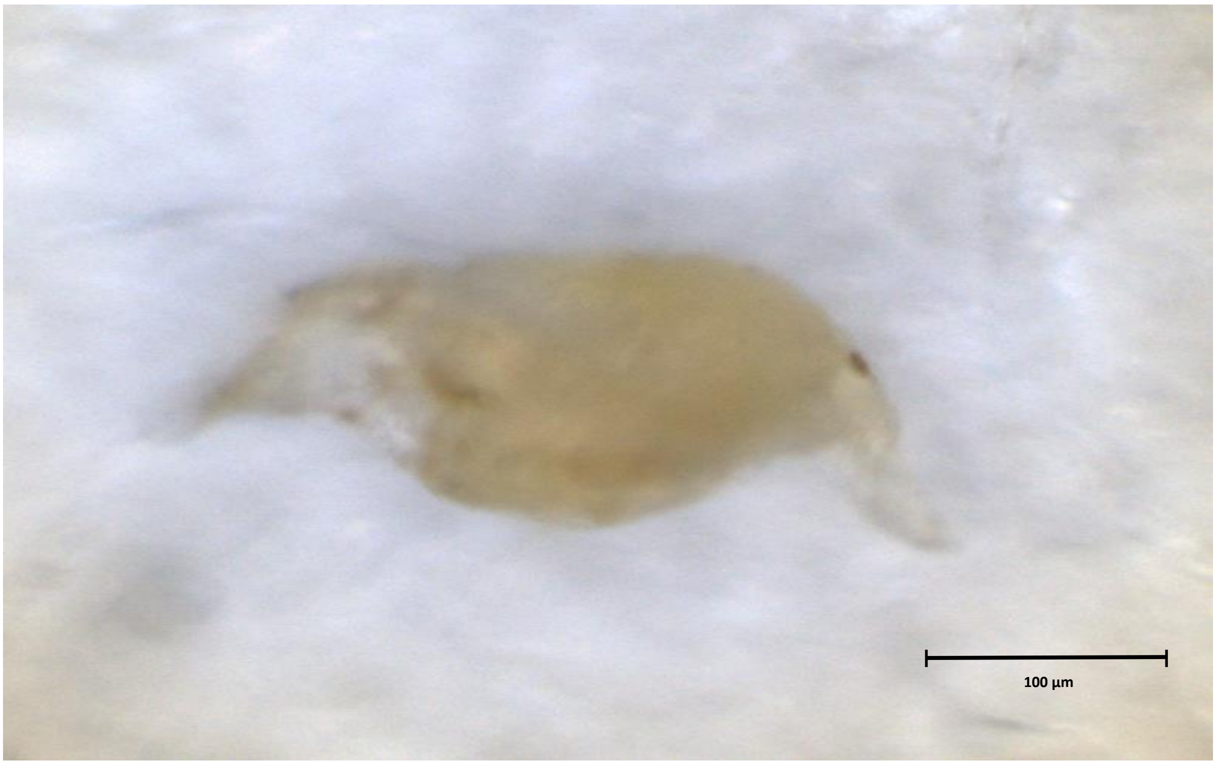

Particle 9. Fragment, 587.74 µm, PE.

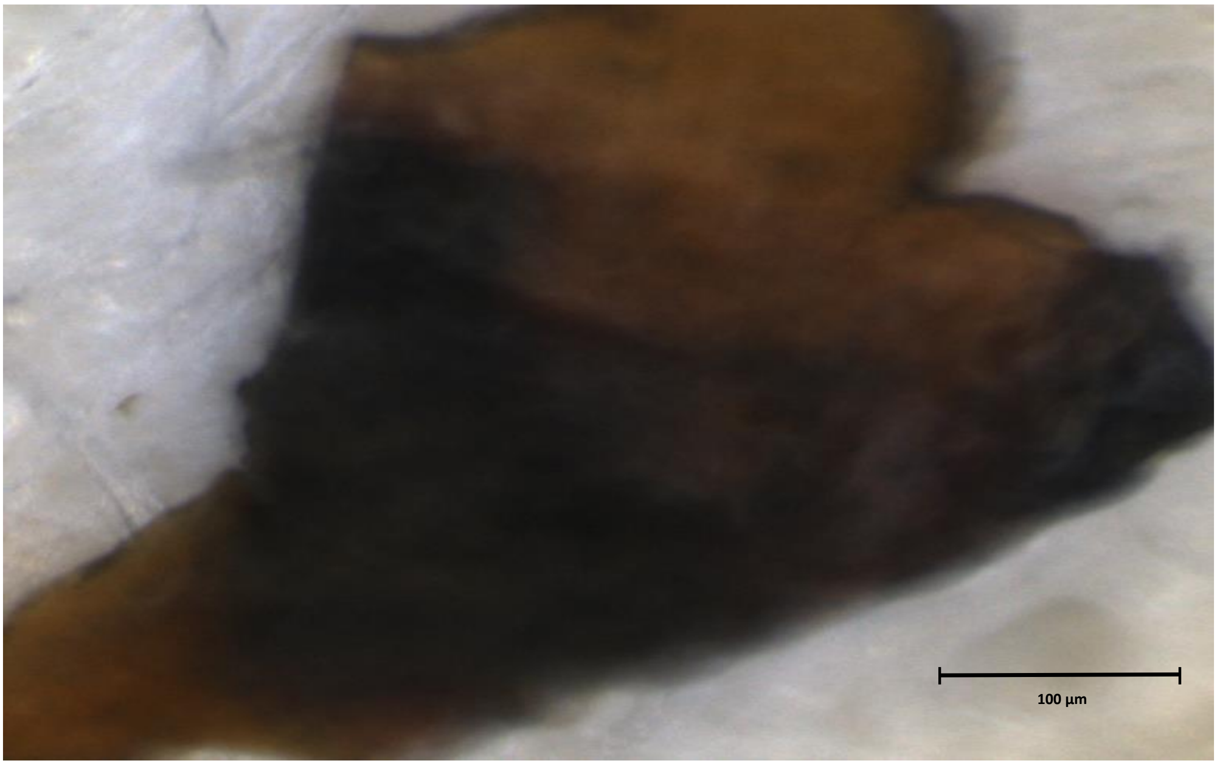

Particle 10. Fragment, 264.75 µm, PP.

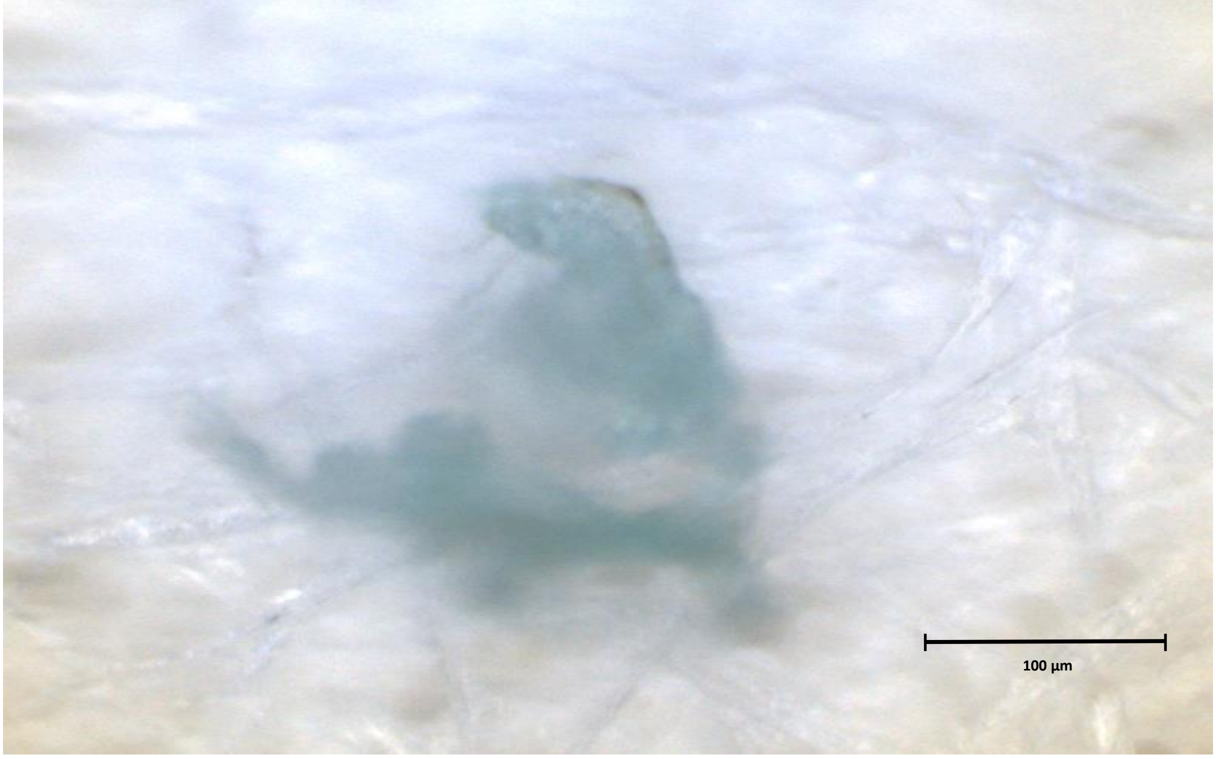

Particle 11. Fragment, 283.58 μm, PP.

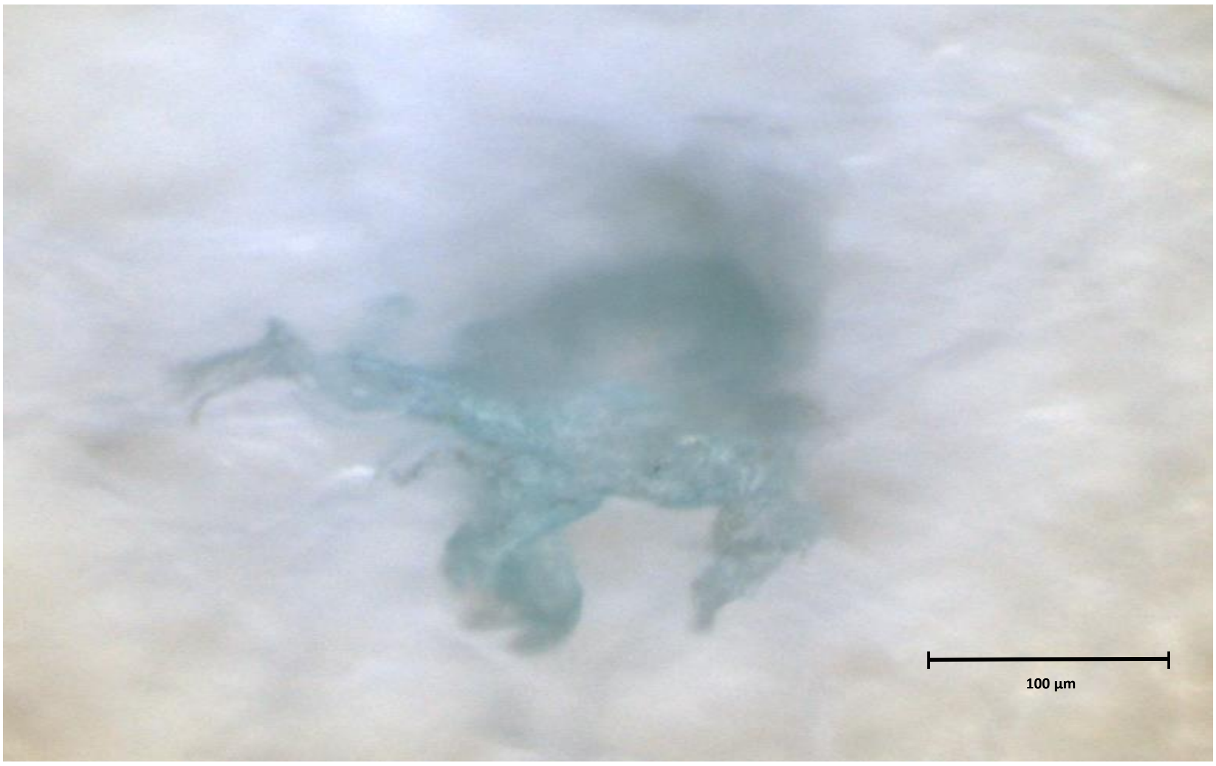

Particle 12. Fragment, 200.30 μm, PE.

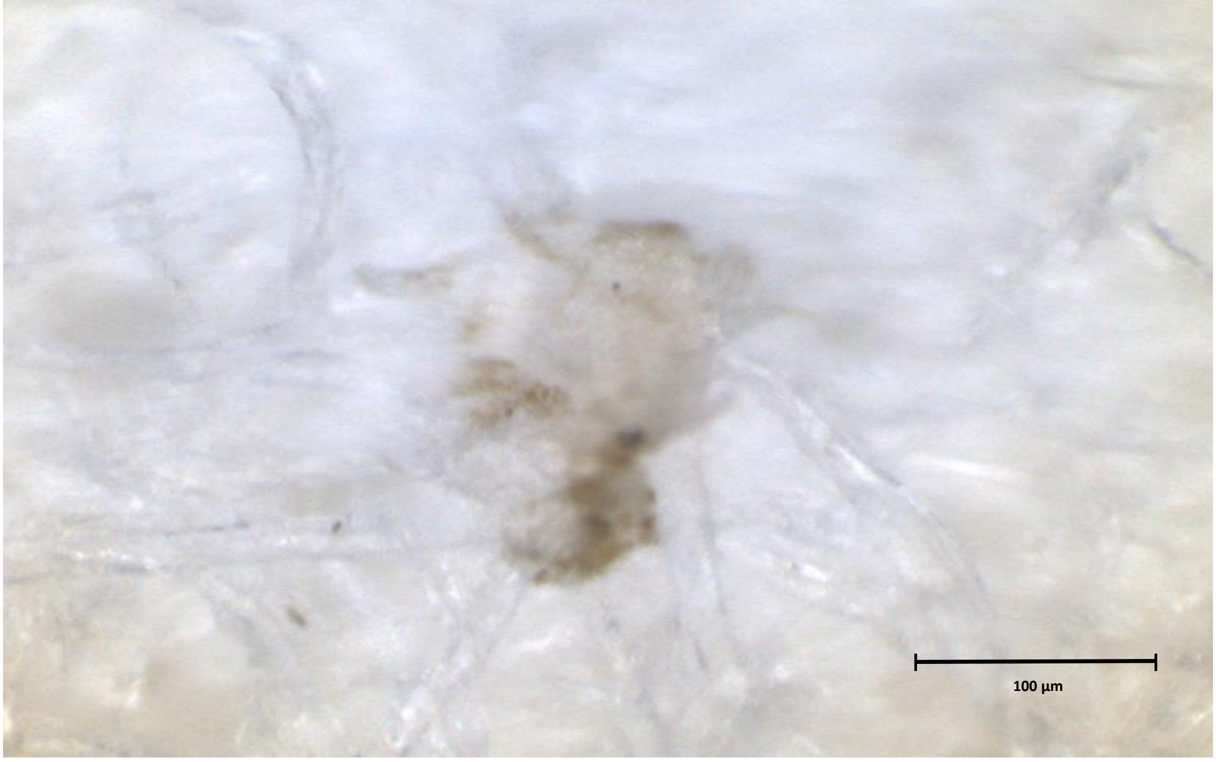

Salt 5

Particle 13. Fragment, 199.91 µm, PP.

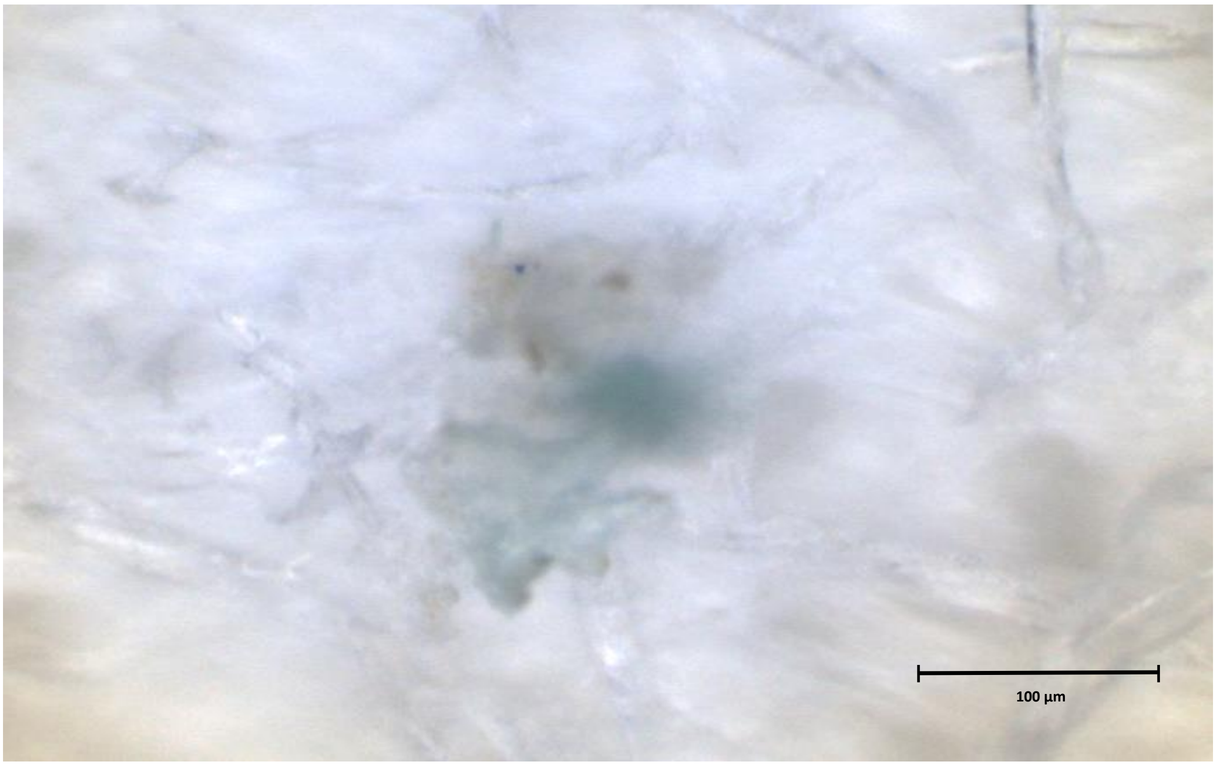

Particle 14. Fragment, 194.61 µm, PP.

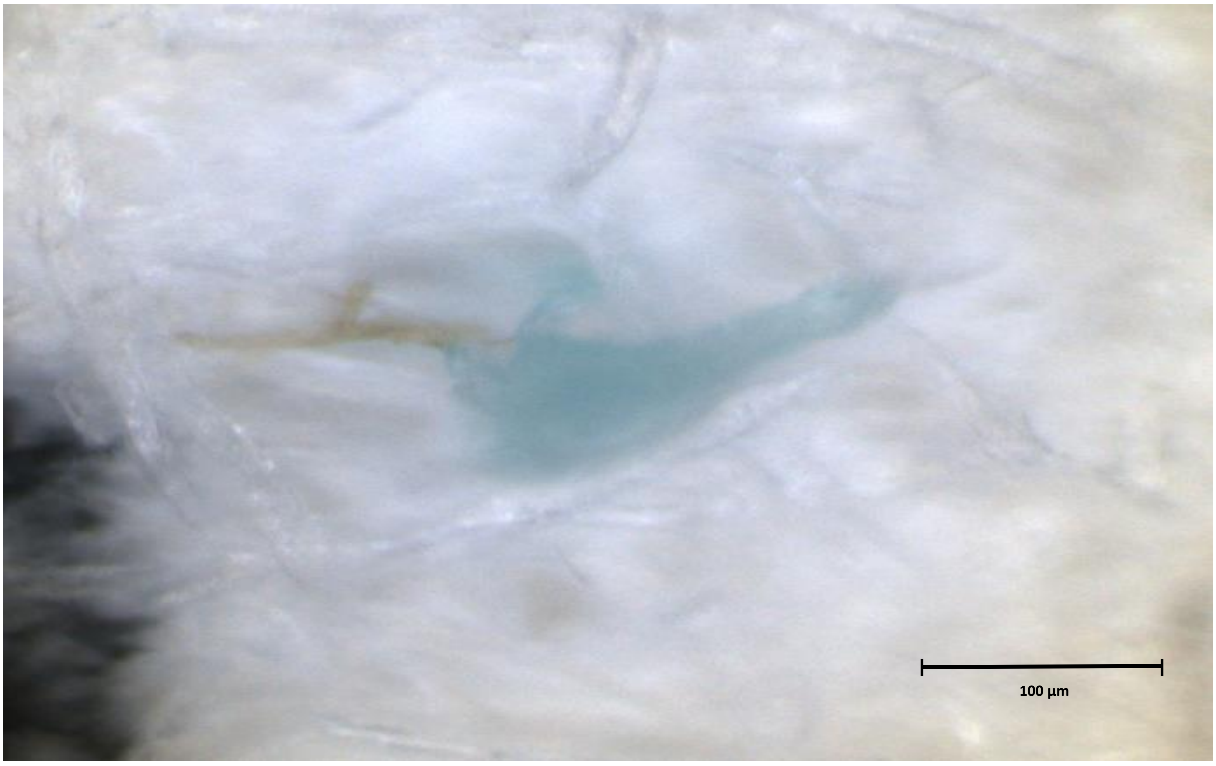

Salt 6

Particle 15. Fragment, 224.01 µm, PP.

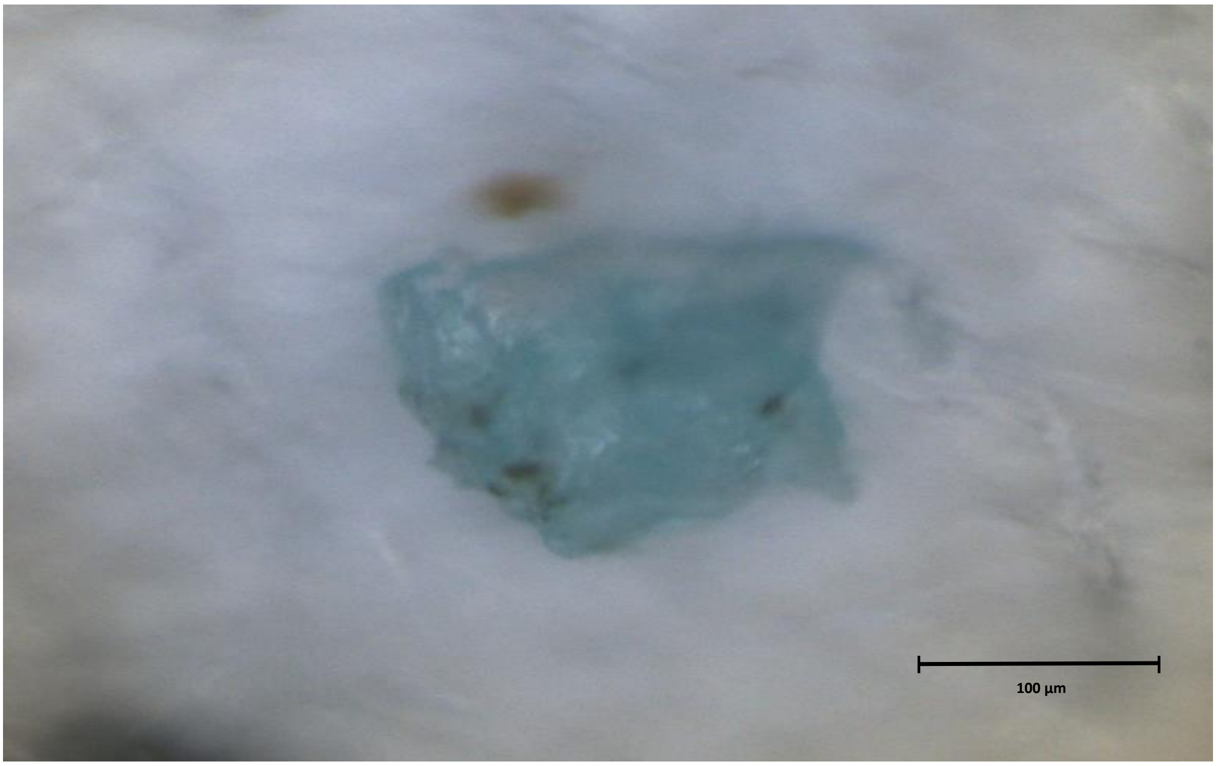

Particle 16. Fragment, 548.49 µm, PE.

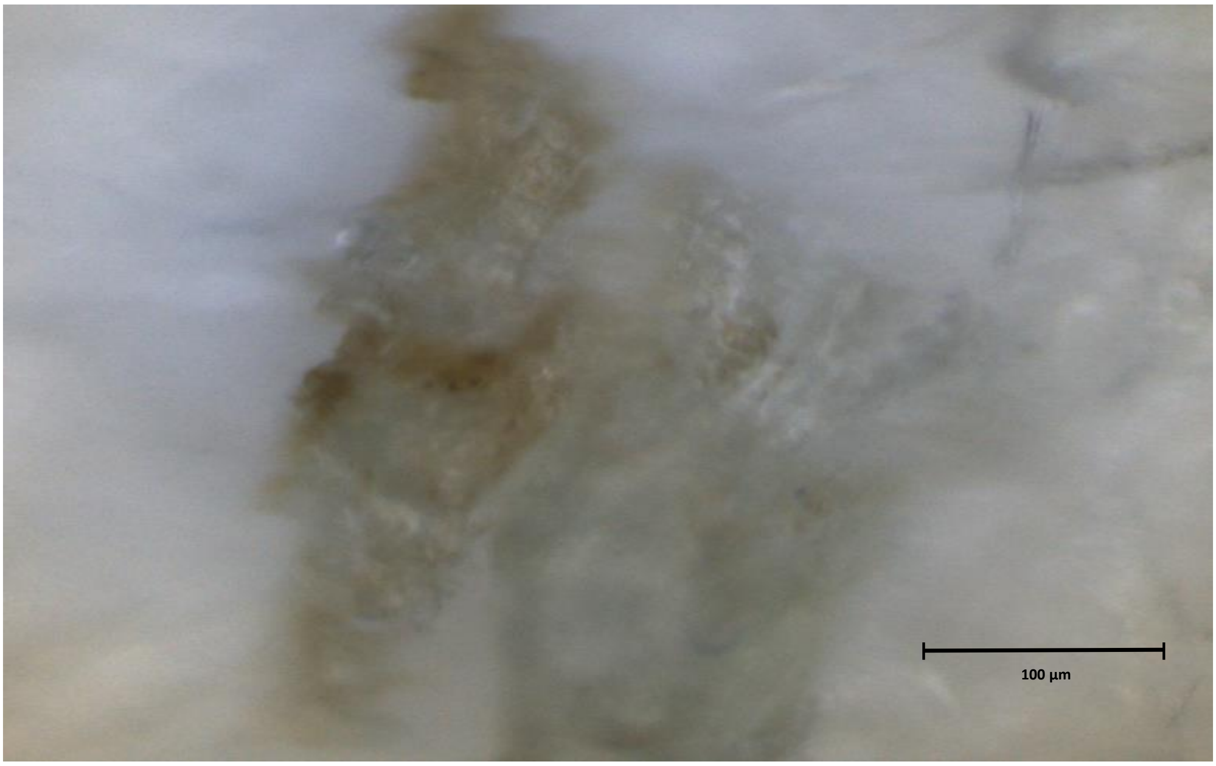

Particle 17. Fragment, 406.72 µm, PE.

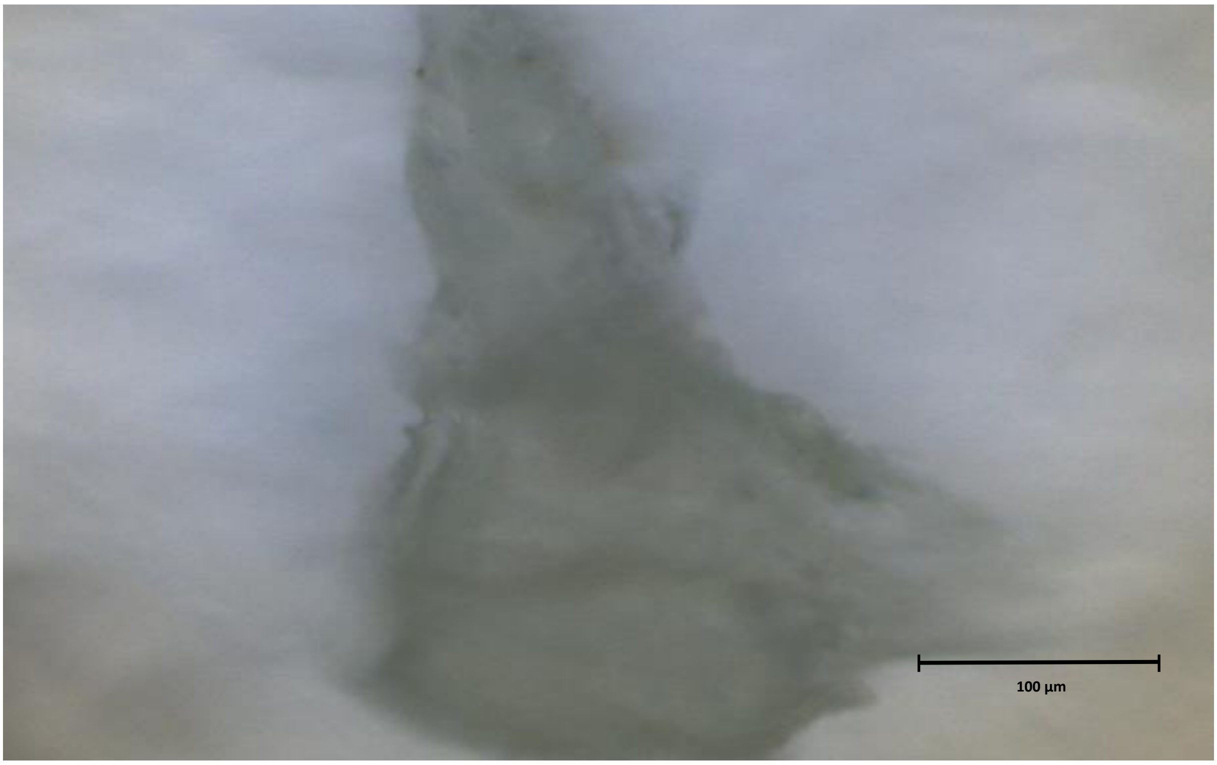

Particle 18. Fragment, 195.00 µm, PP.

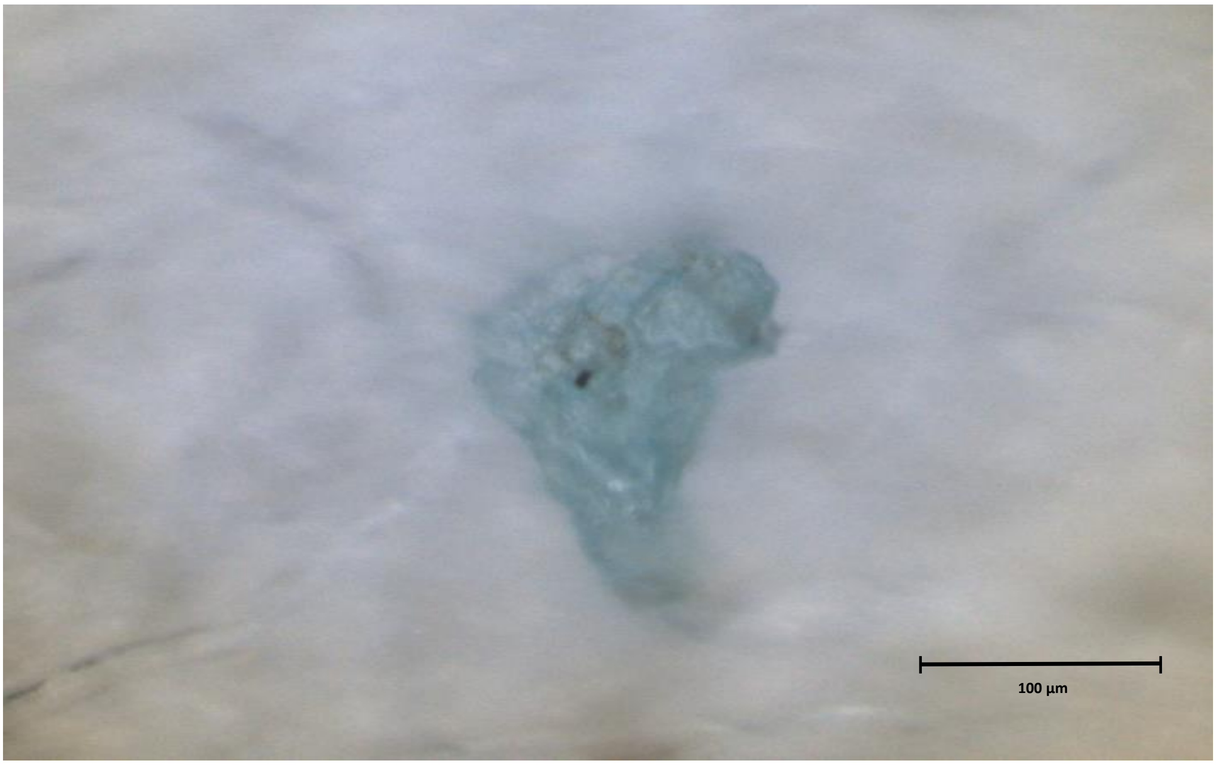

Particle 19. Fragment, 151.87 µm, PP.

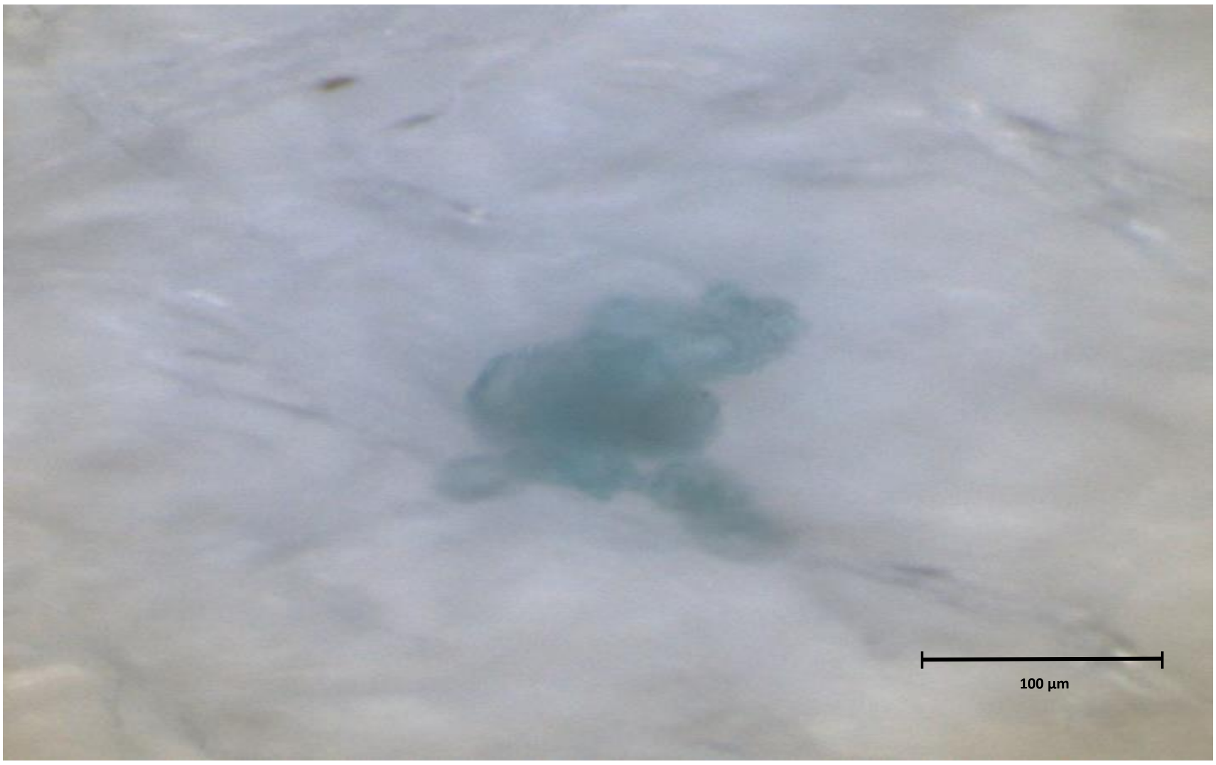

Particle 20. Fragment, 693.91 µm, PP.

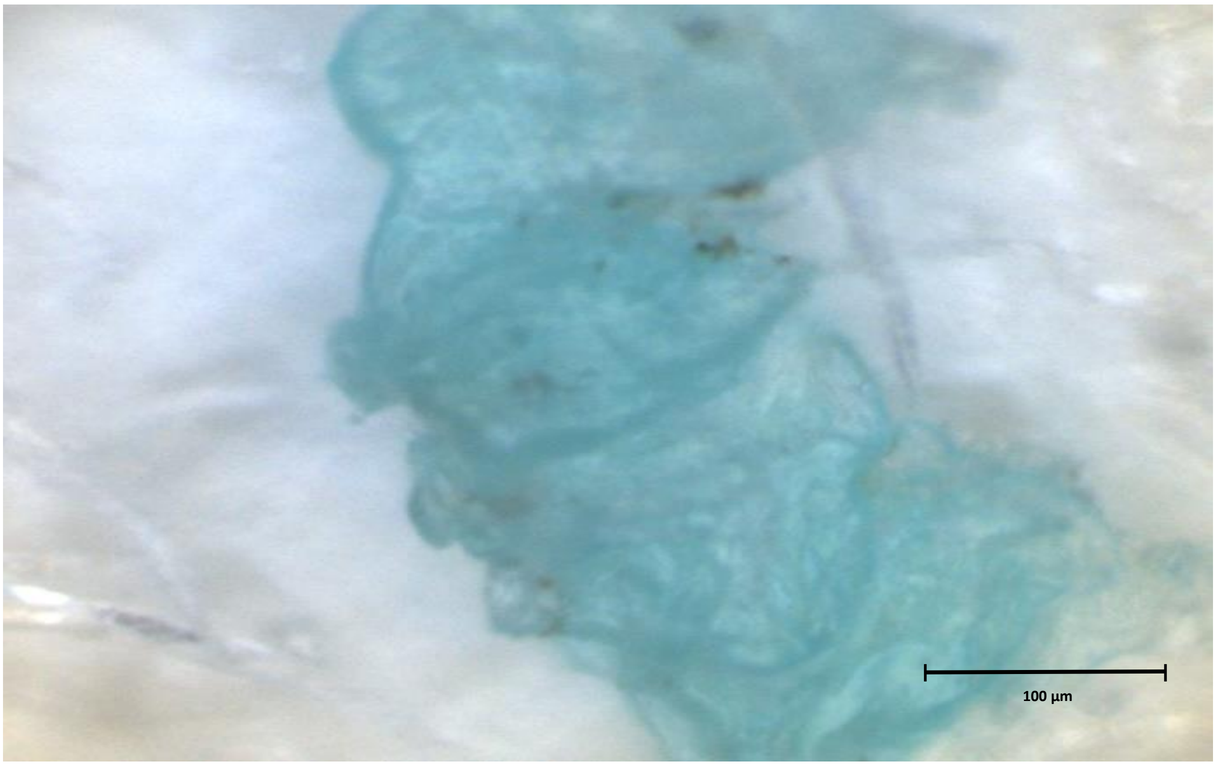

Particle 21. Fragment, 305.30, PE.

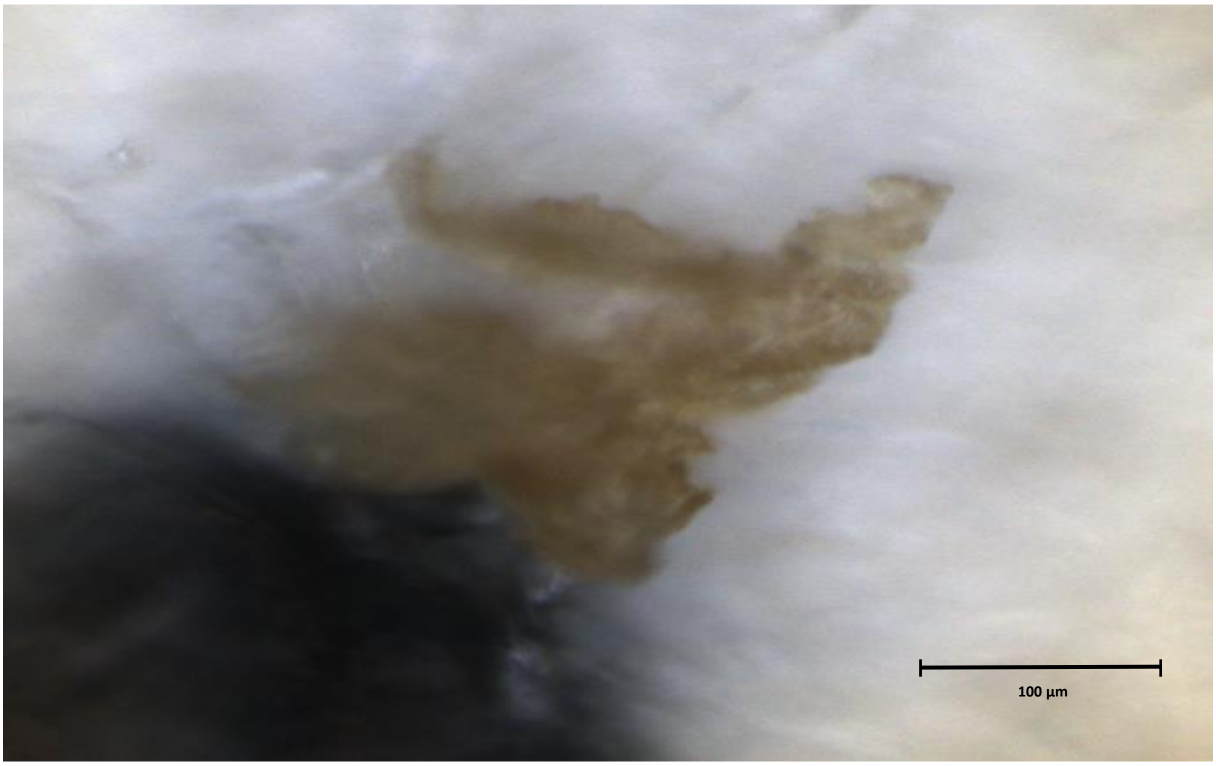

Salt 7

Particle 22. Fragment, 101.86, PES.

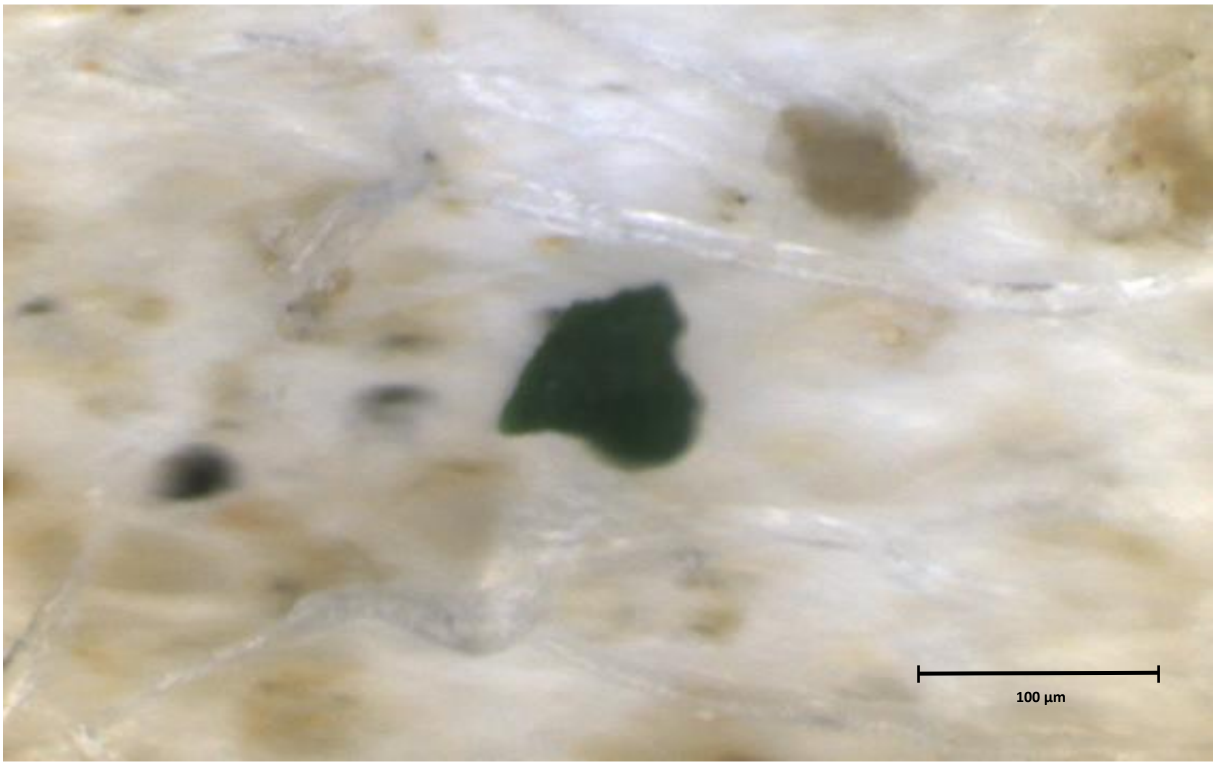

Particle 23. Fragment, 89.72, PP.

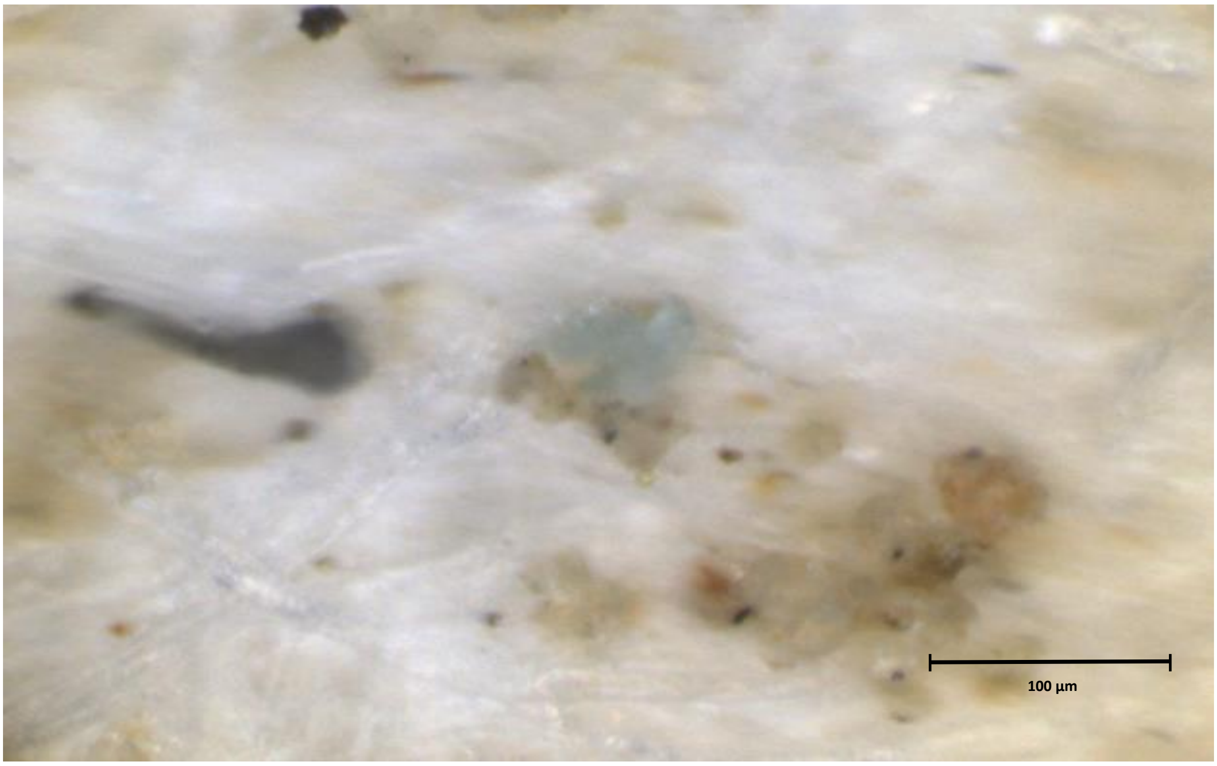

Particle 24. Fragment, 1474.88 μm, PE.

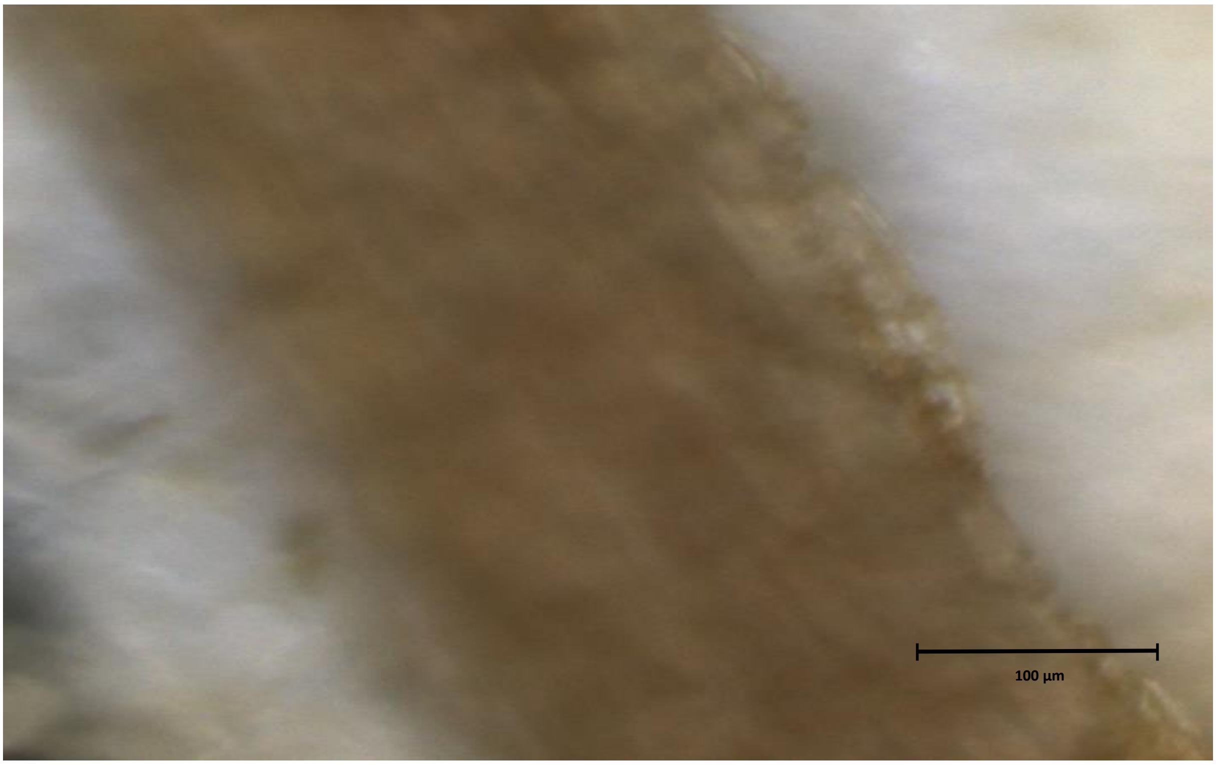

Salt 8

Particle 25. Fragment, 468.67, PE.

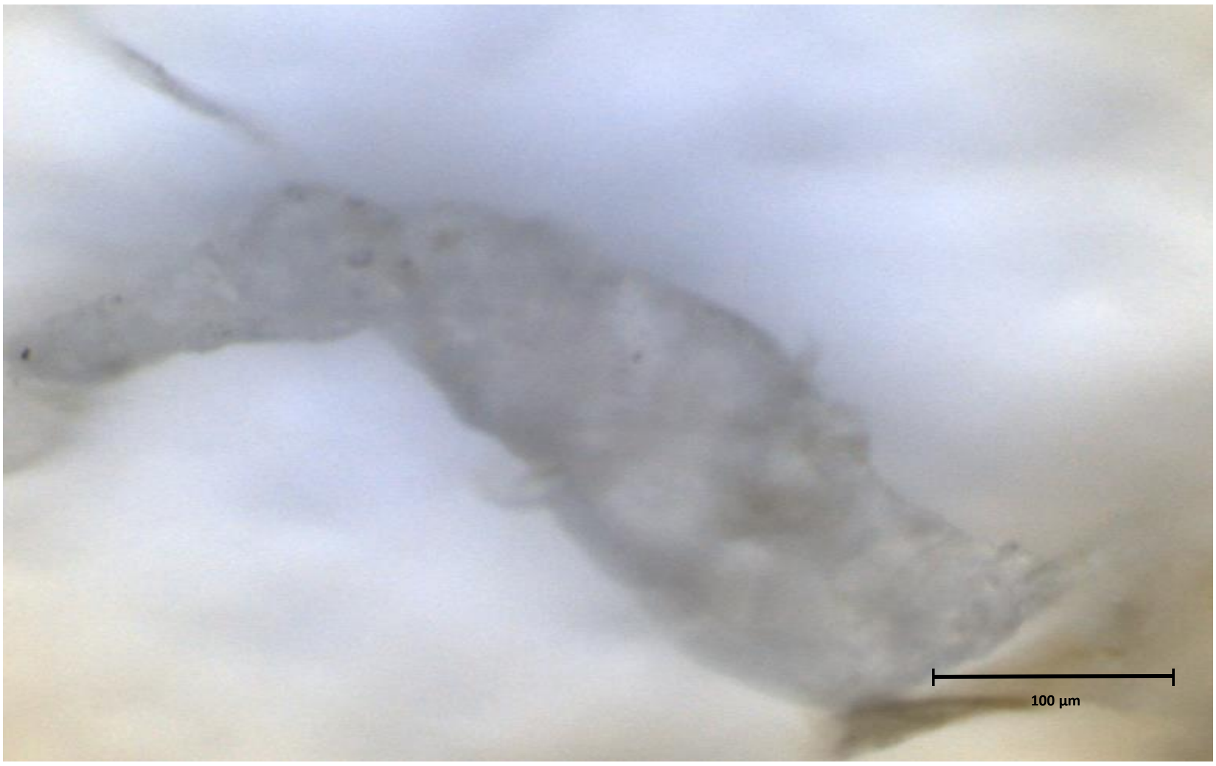

Particle 26. Fragment, 184.12, PE.

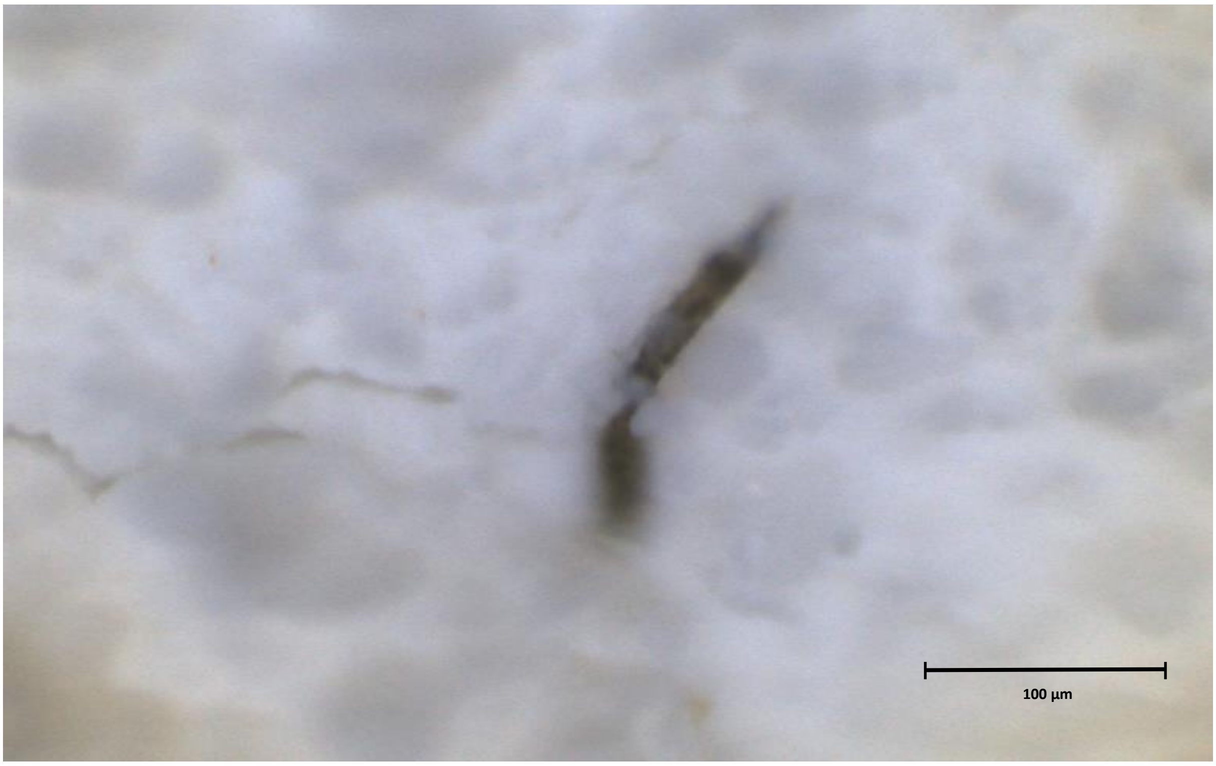

Particle 27. Fragment, 264.58, PP.

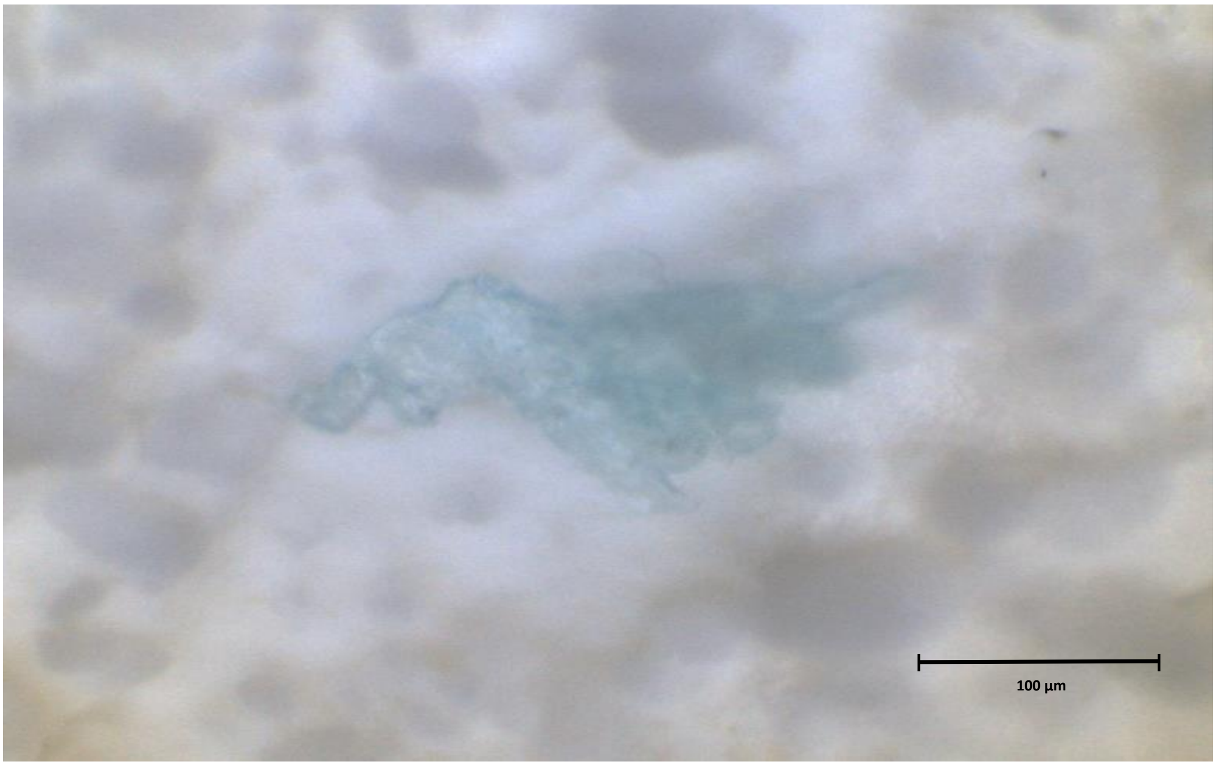

Particle 28. Fragment, 504.39, PE.

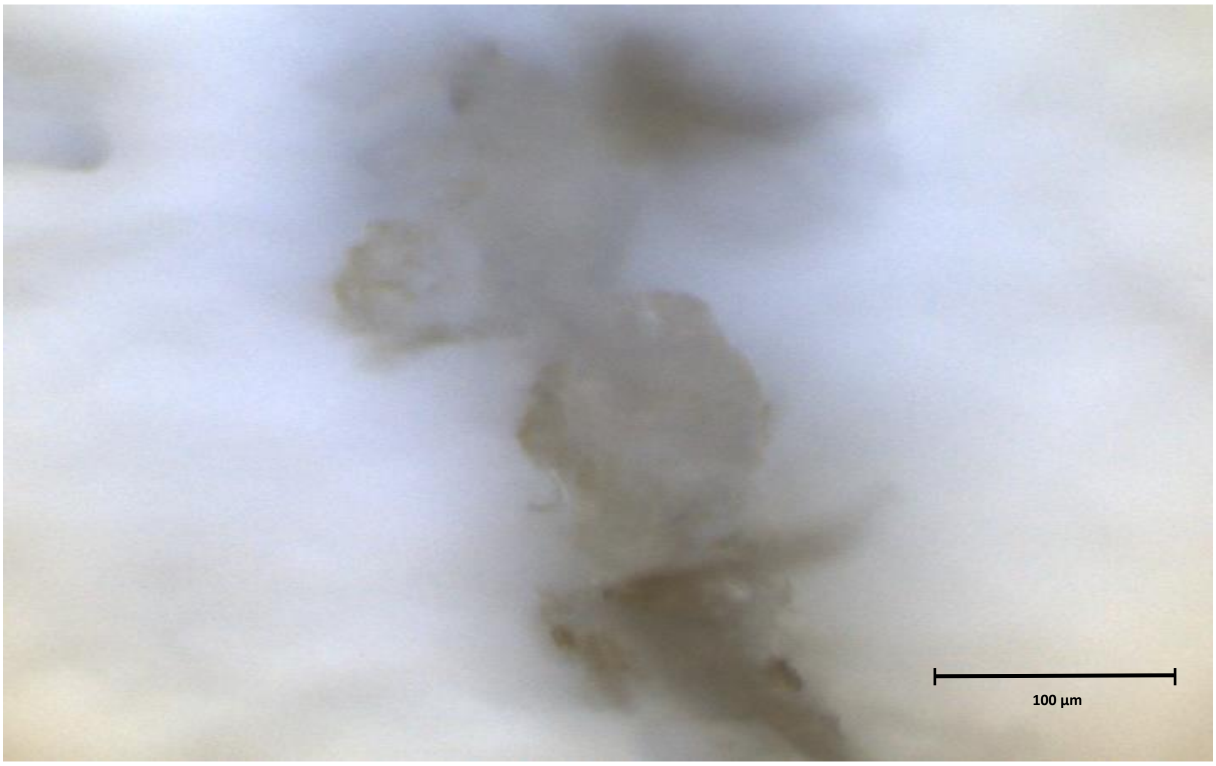

Particle 29. Fragment, 868.87, PE.

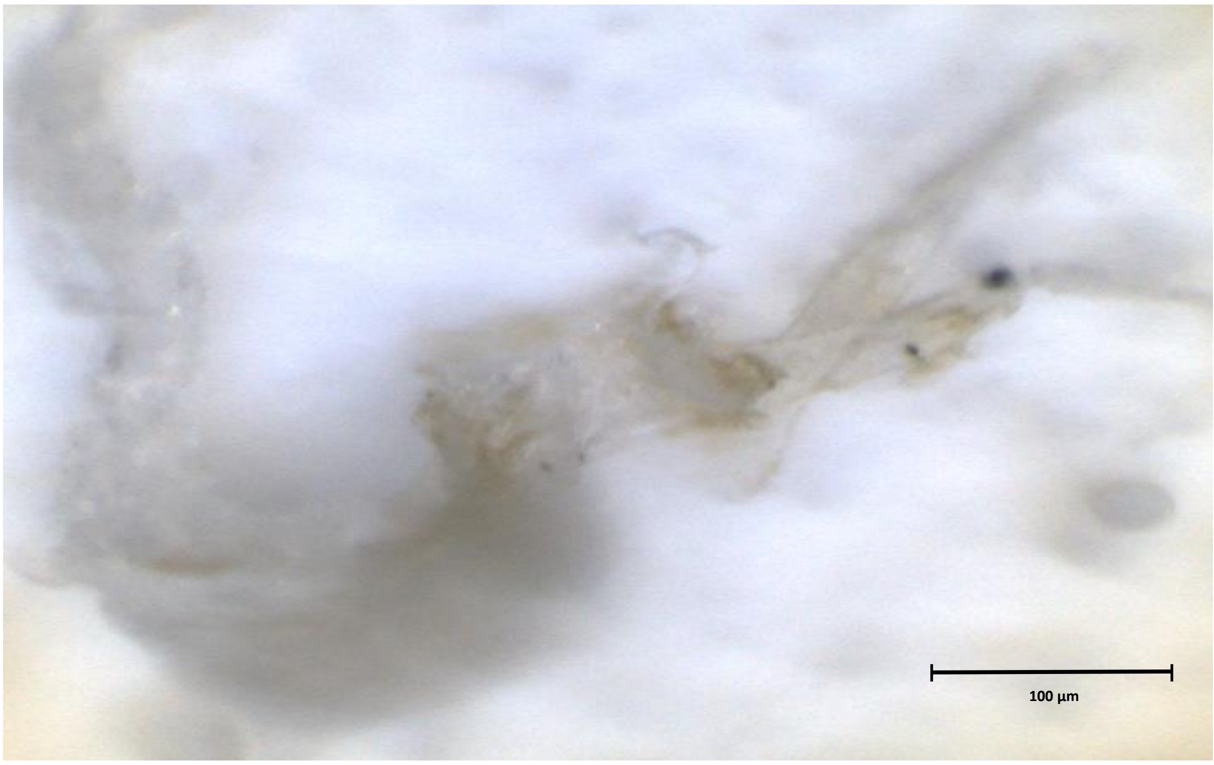

Salt 9

Particle 30. Fragment, 410.36 µm, PS.

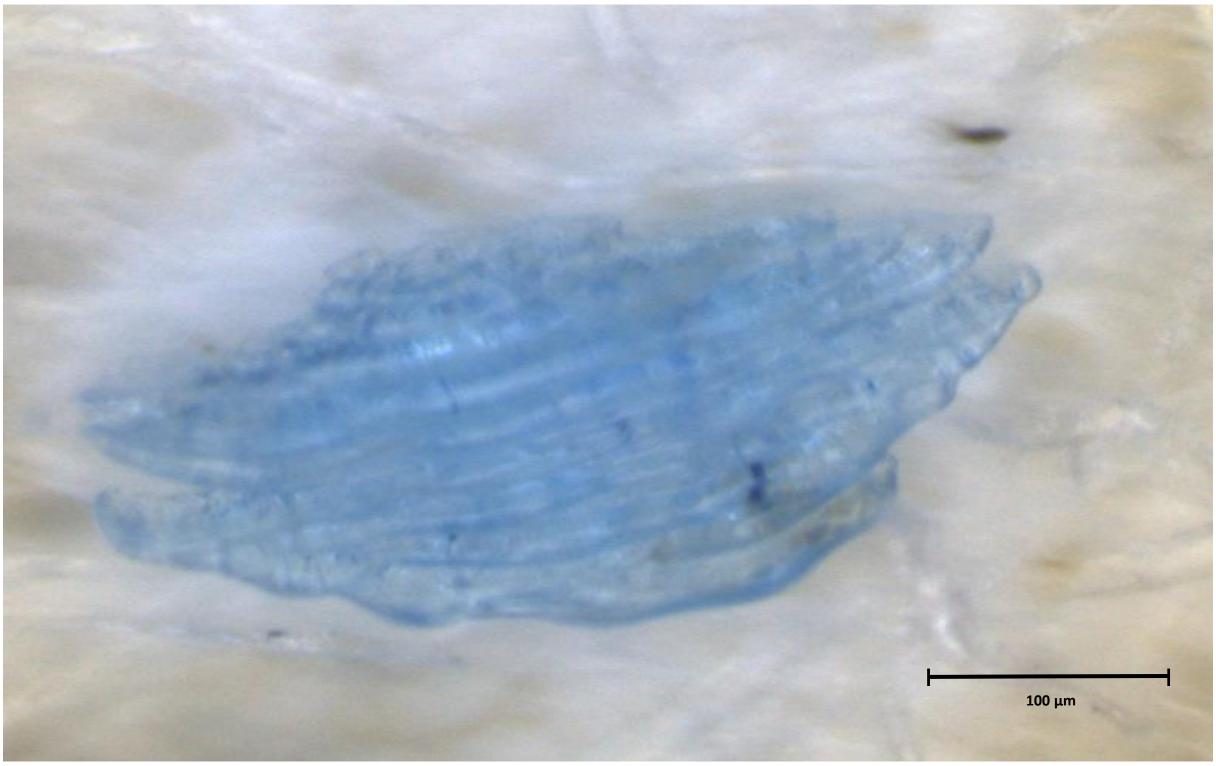

Particle 31. Fragment, 236.60 µm, PS.

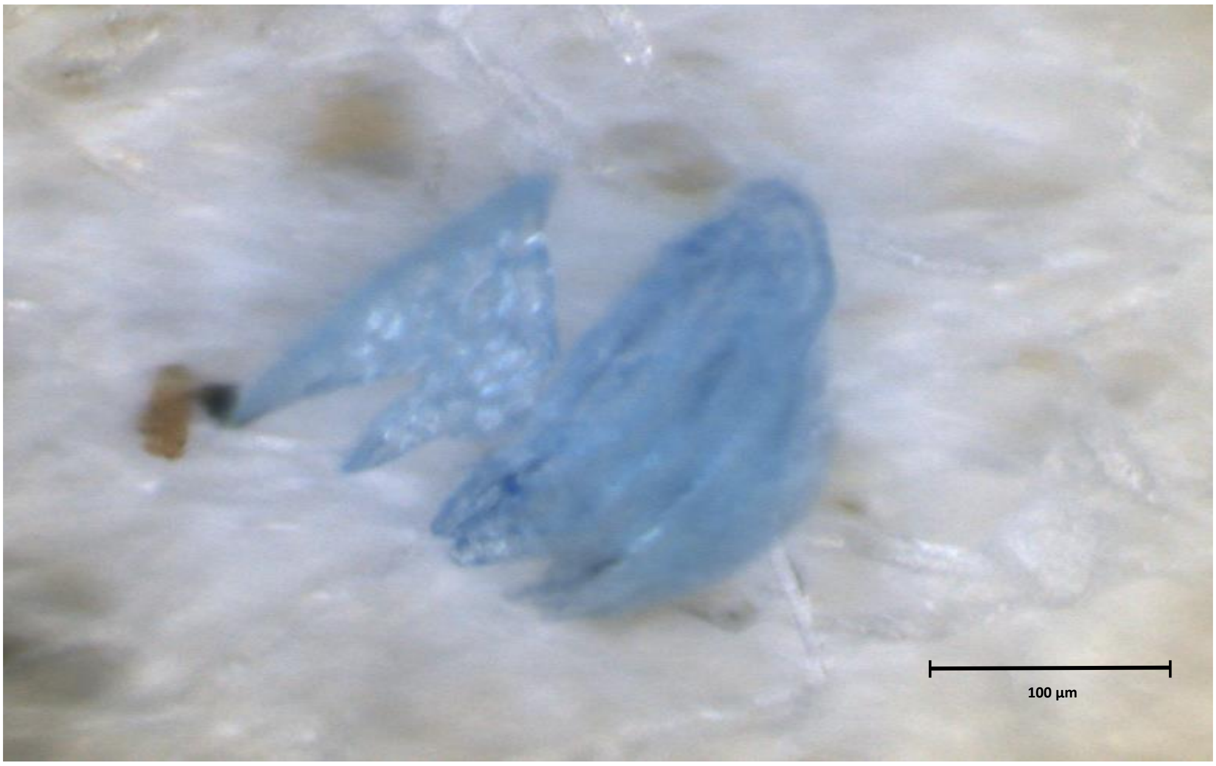

Particle 32. Fragment, 331.03 µm, PS.

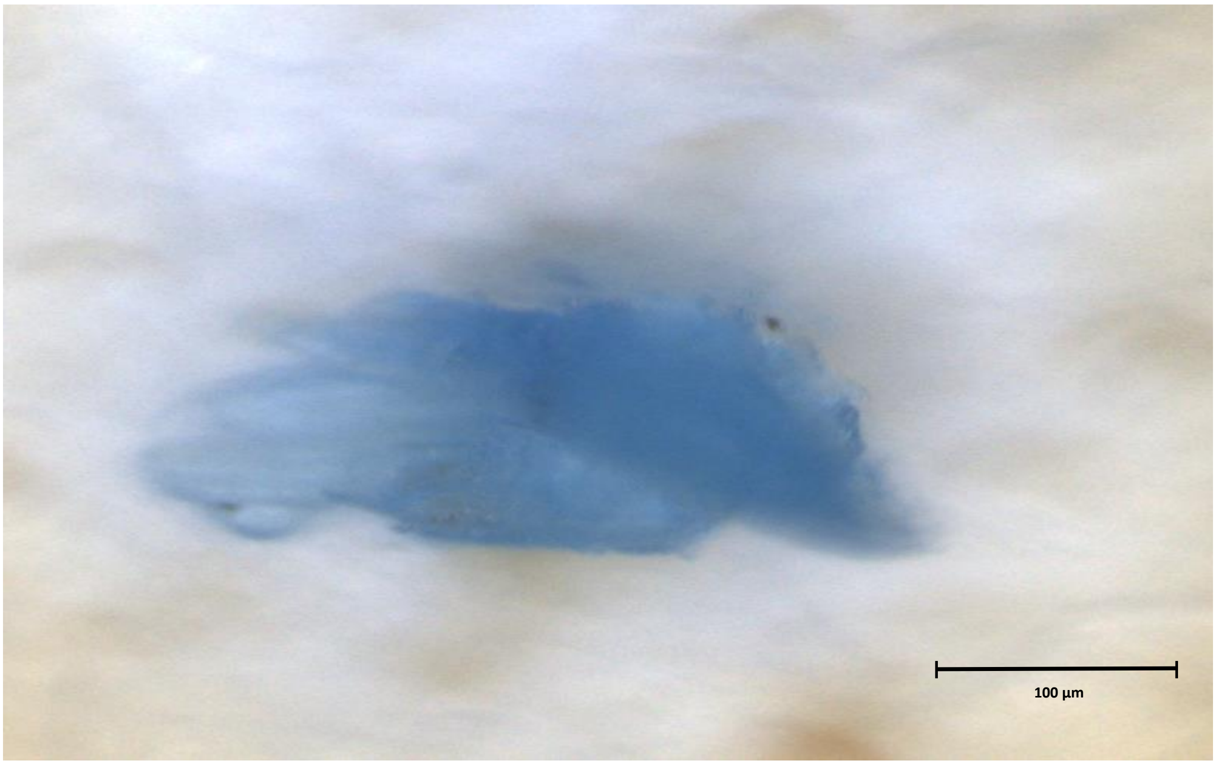

Particle 33. Fragment, 580.92 µm, PS.

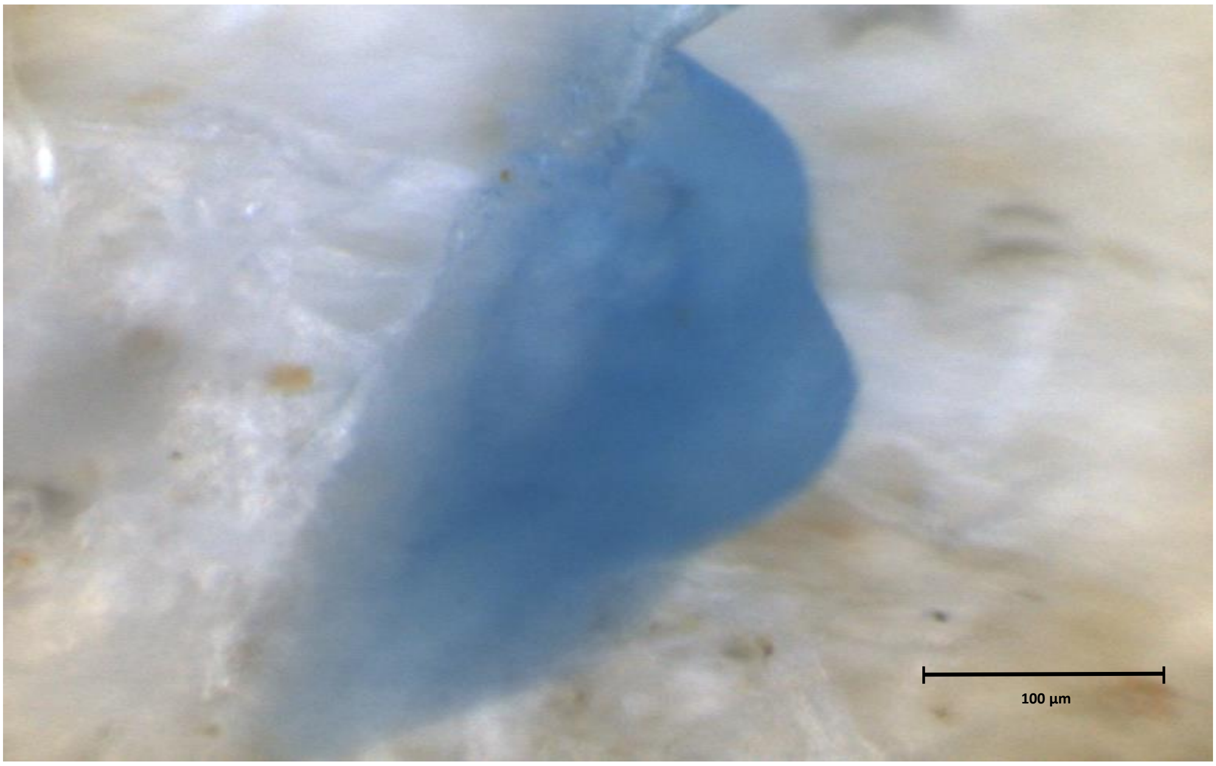

Particle 34. Fragment, 377.92 μm, PS.

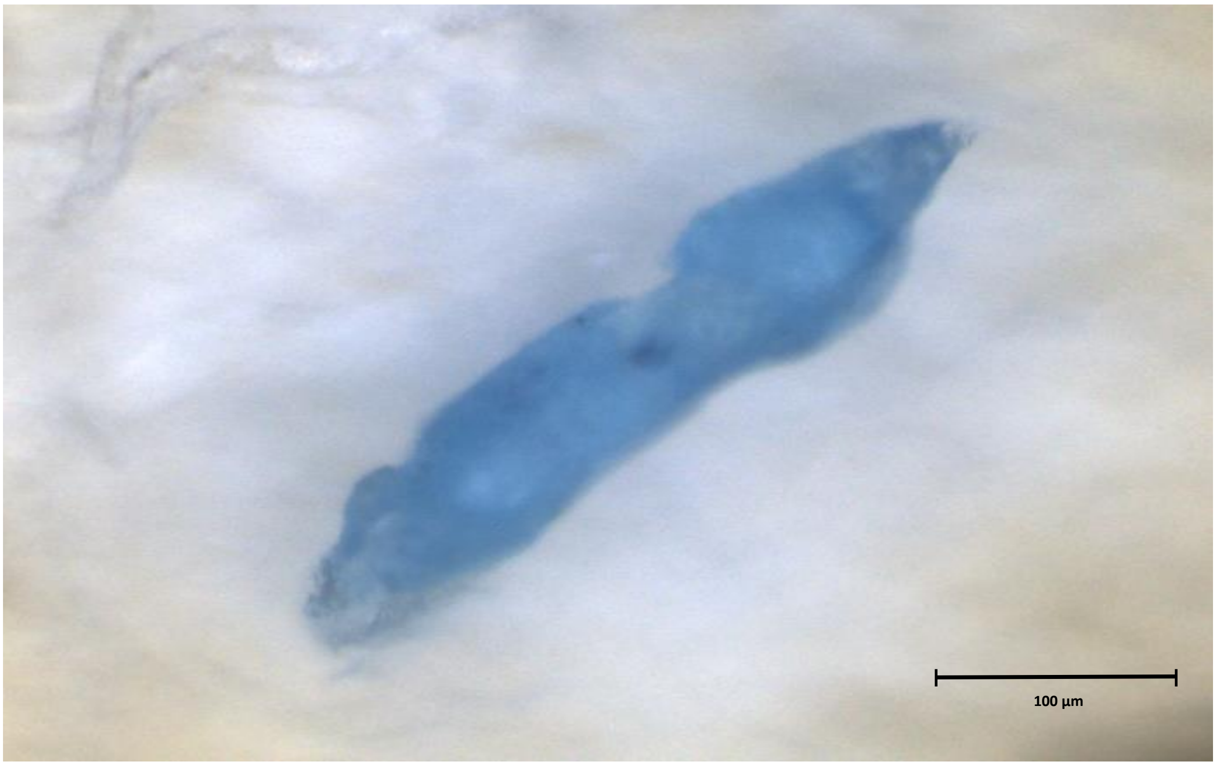

Salt 10

Particle 35. Fragment, 367.86 µm, PAC.

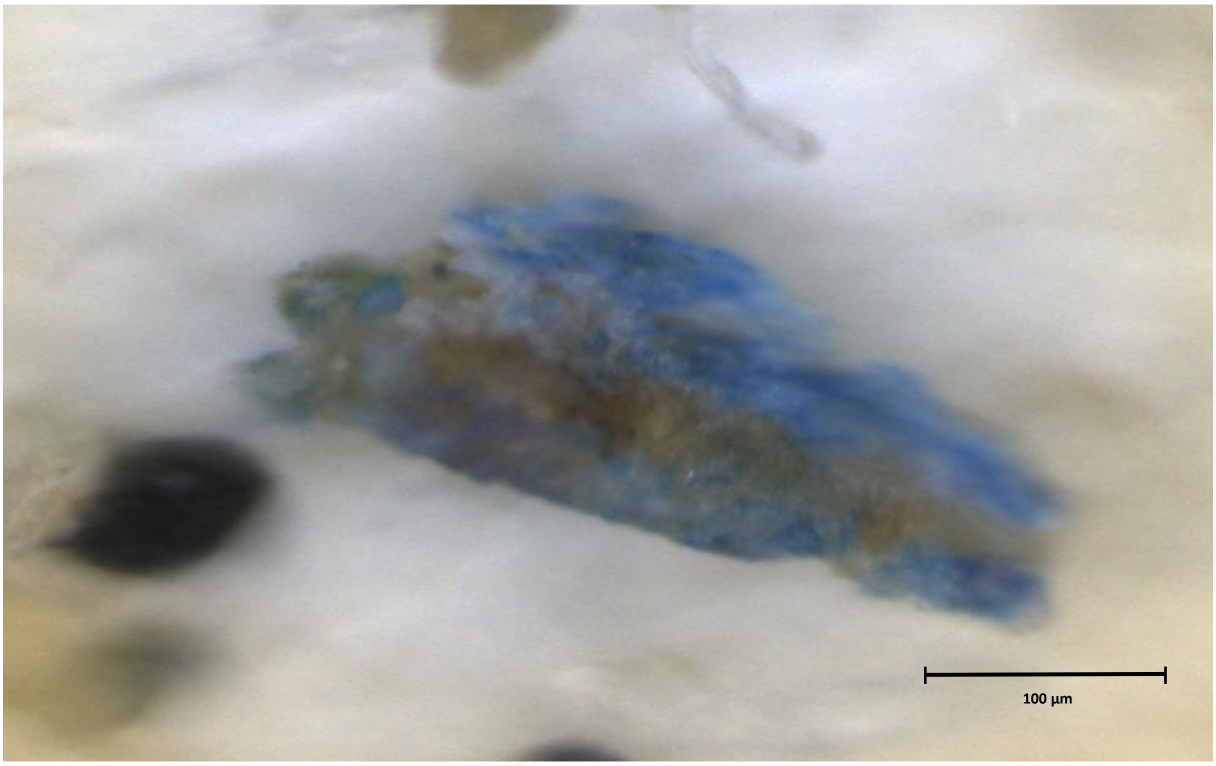

Particle 36. Fragment, 313.74 µm, PE.

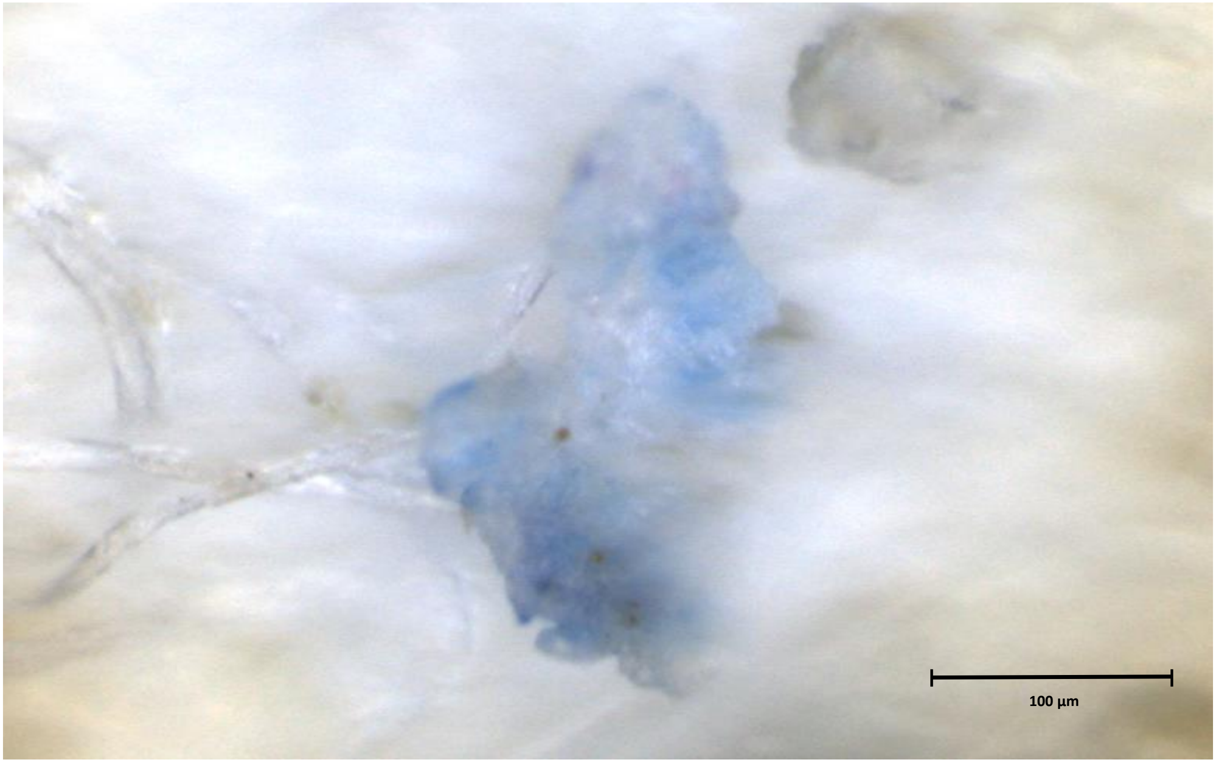

Particle 37. Fragment, 141.63 µm, PS.

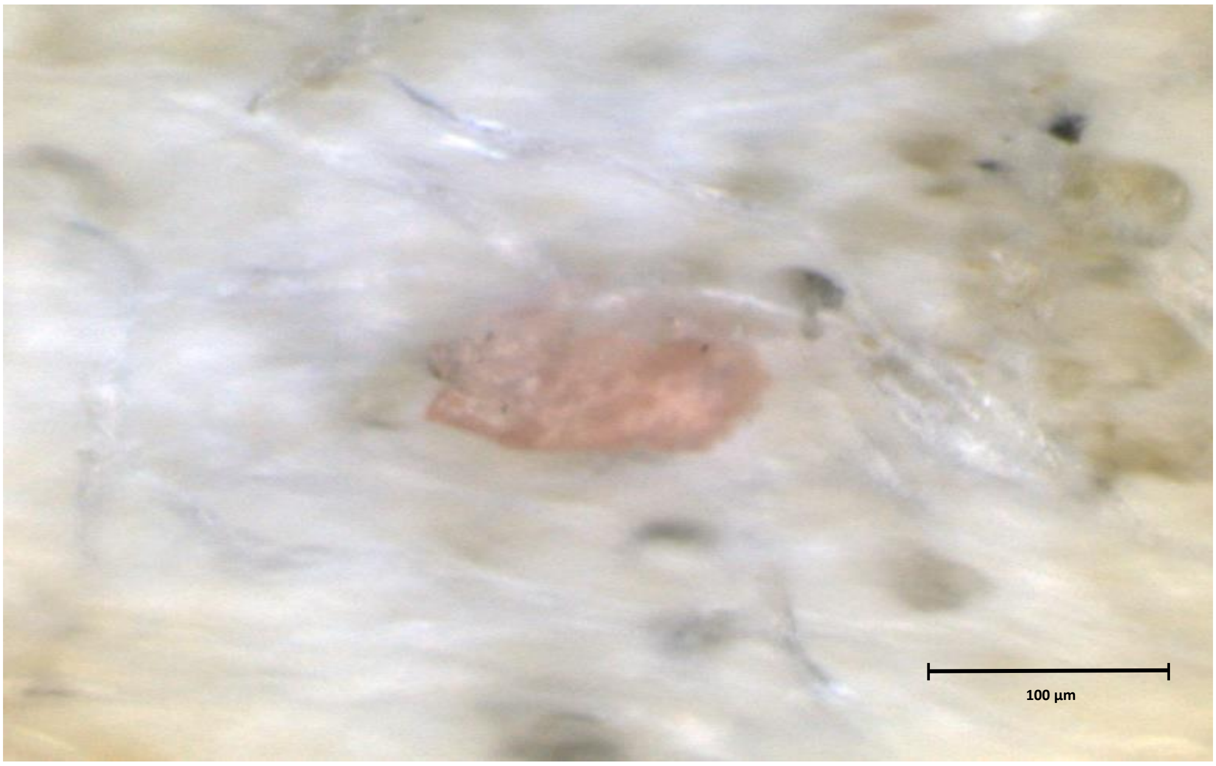

Particle 38. Fragment, 206.45 µm, PE.

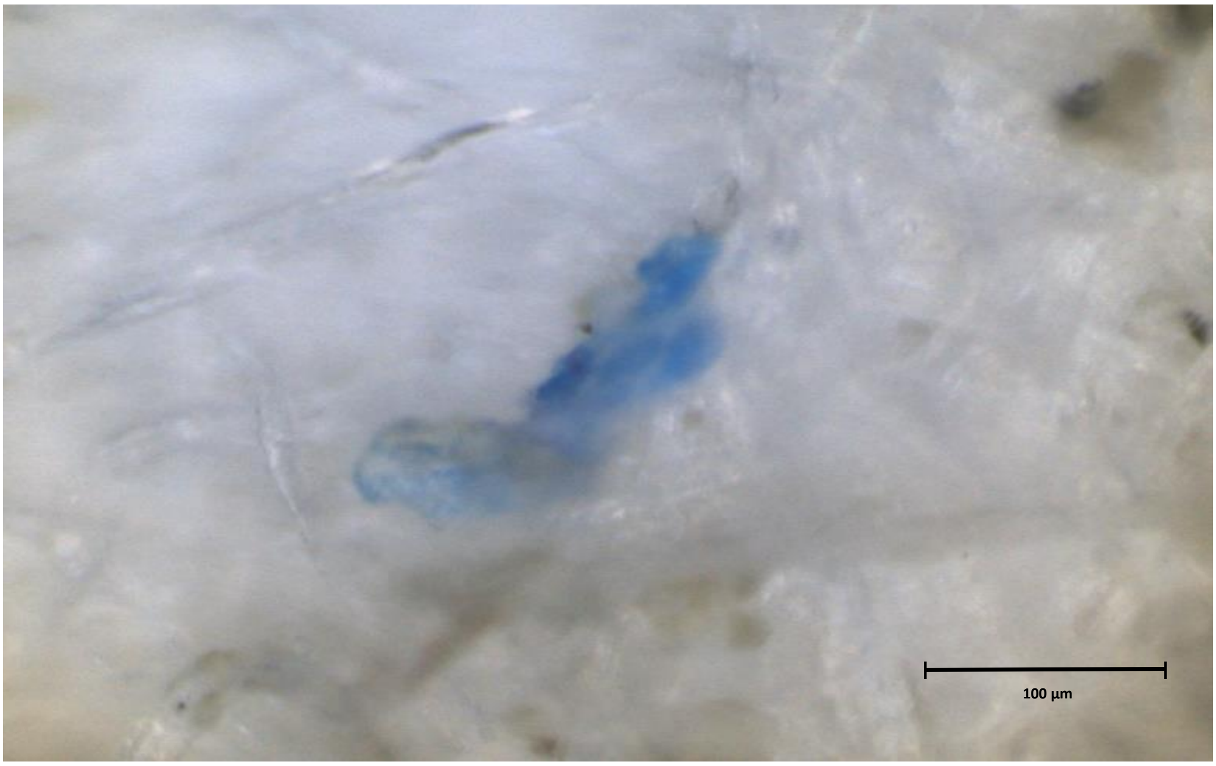

Salt 11

Particle 39. Fragment, 713.06 µm, PE.

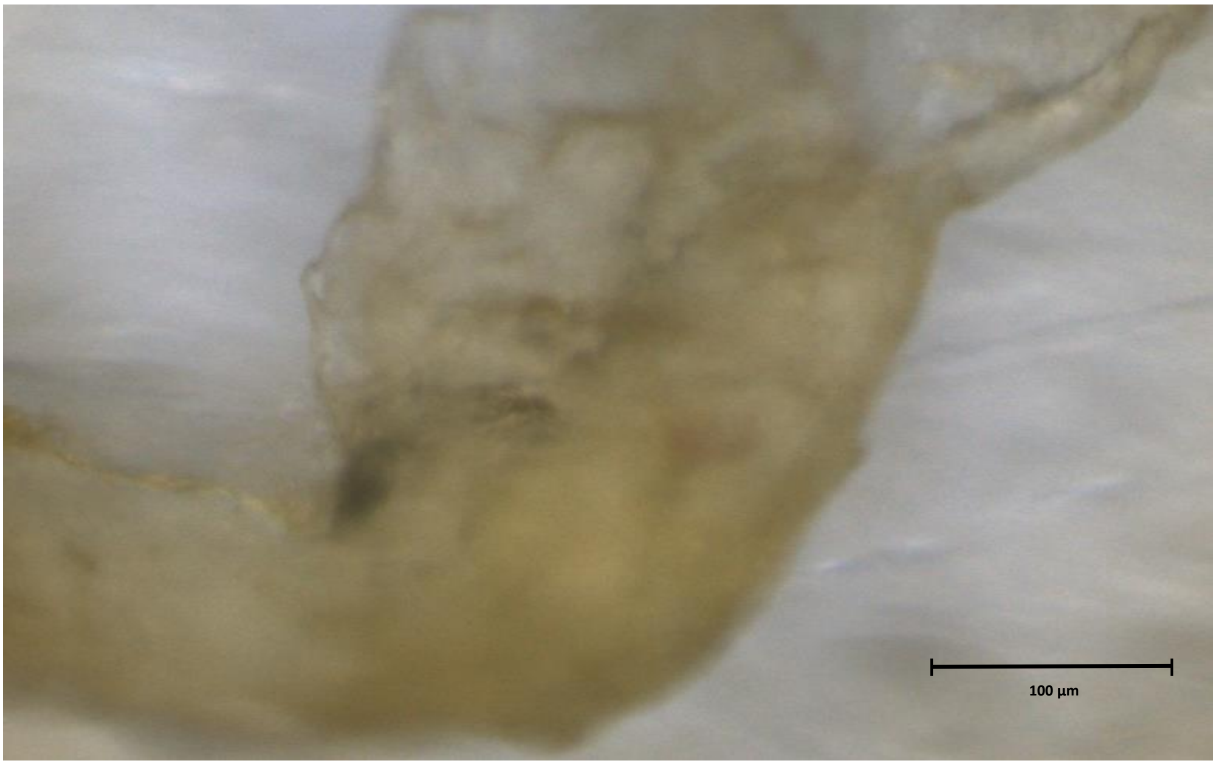

Particle 40. Fragment, 398.46 µm, PP.

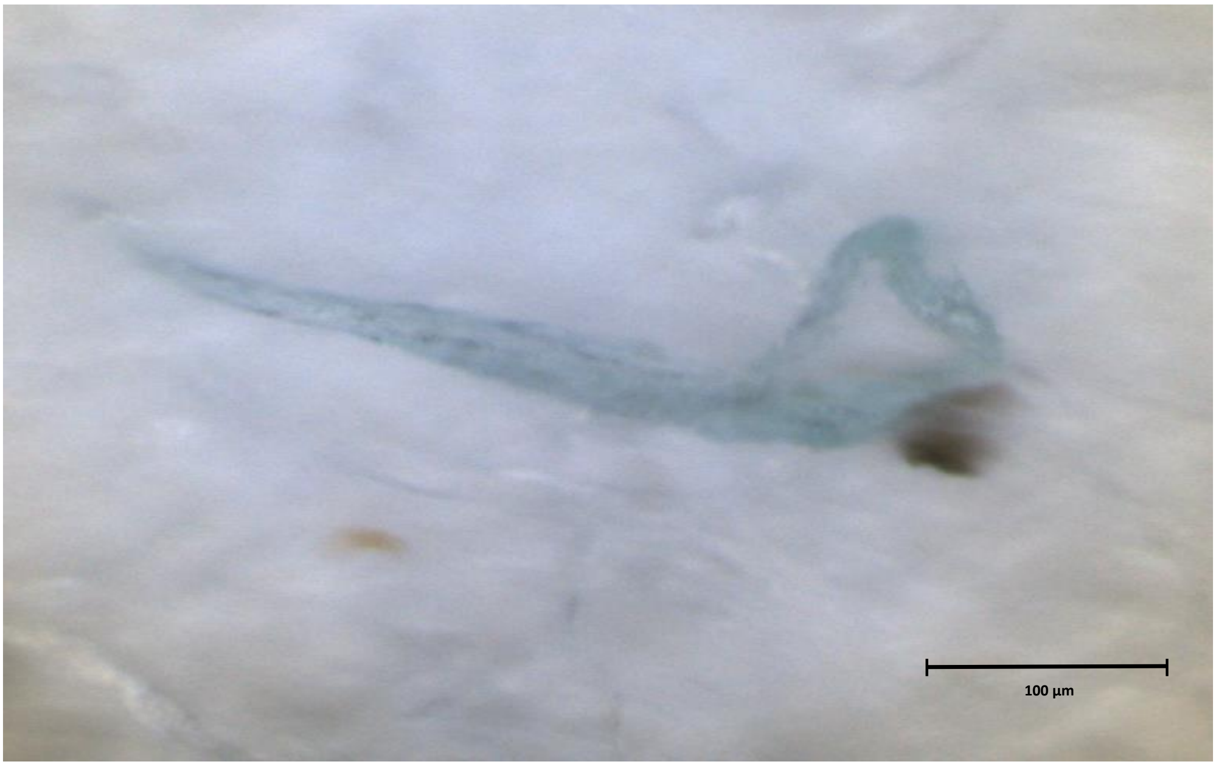

Particle 41. Fragment, 150.42 µm, PP.

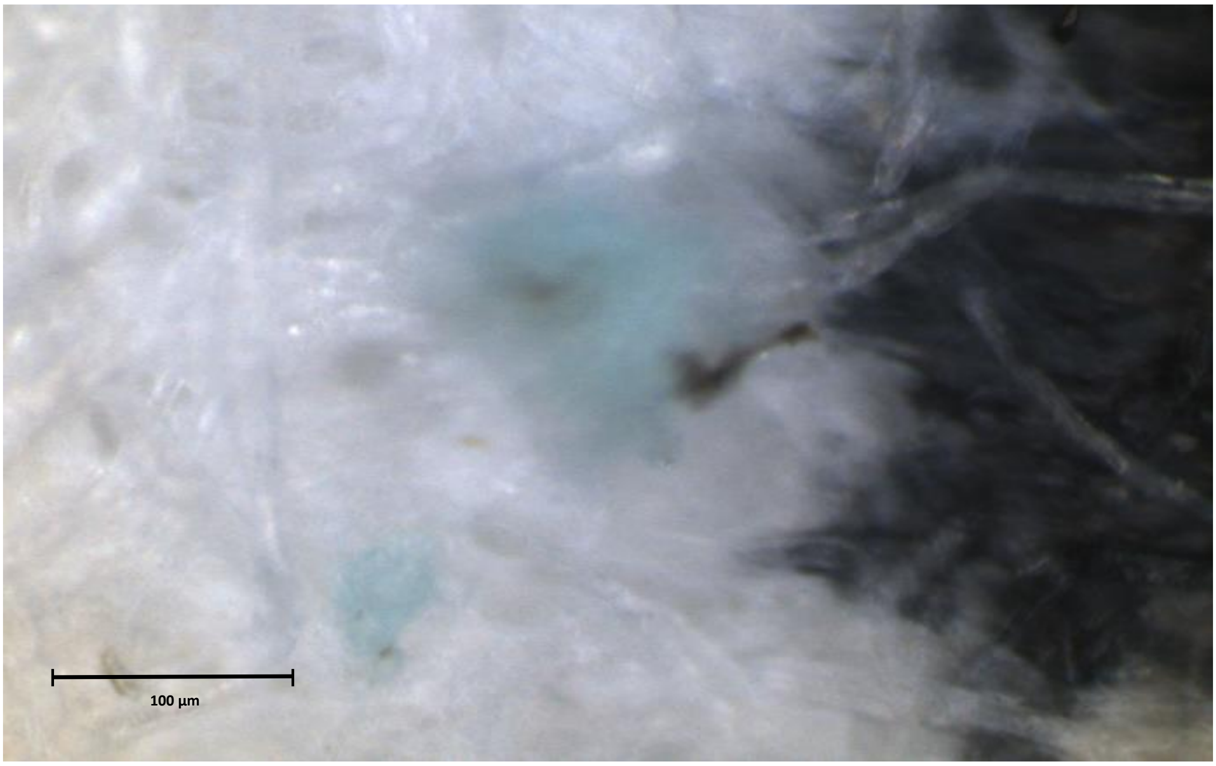

Particle 42. Fragment, 216.03 µm, PP.

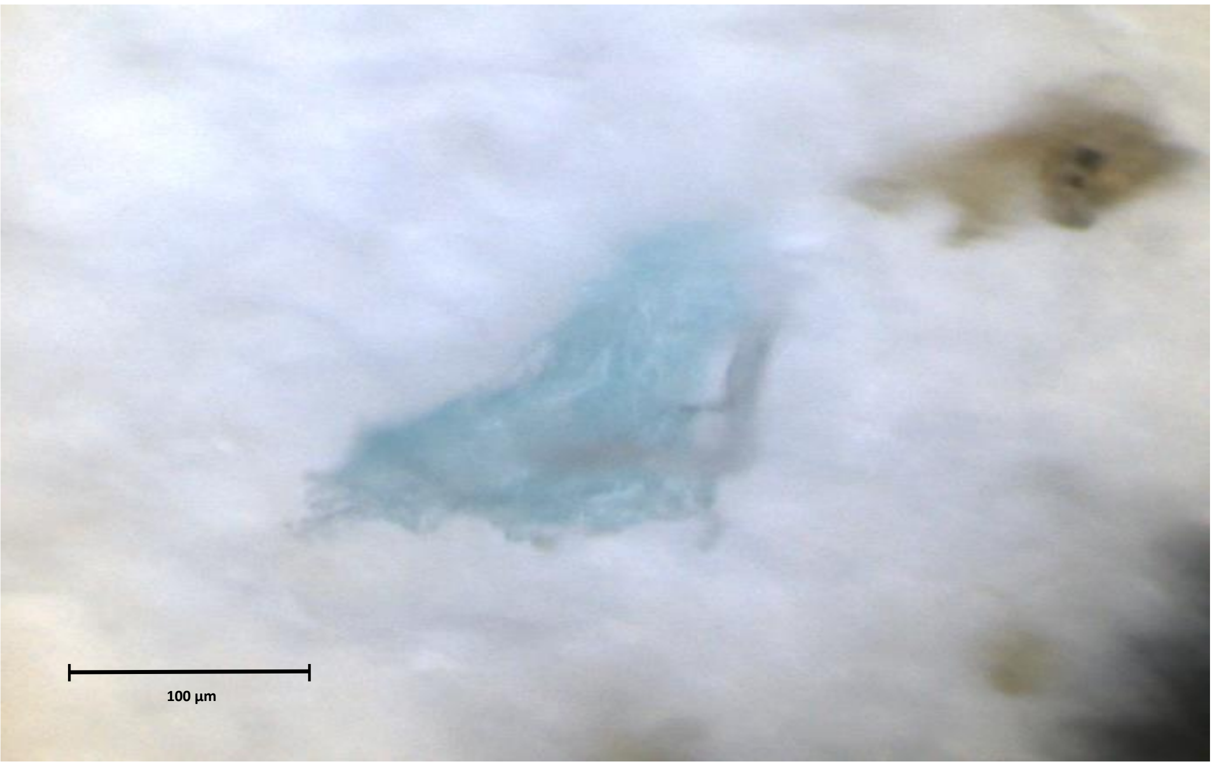

Particle 43. Fragment, 180.97, PP.

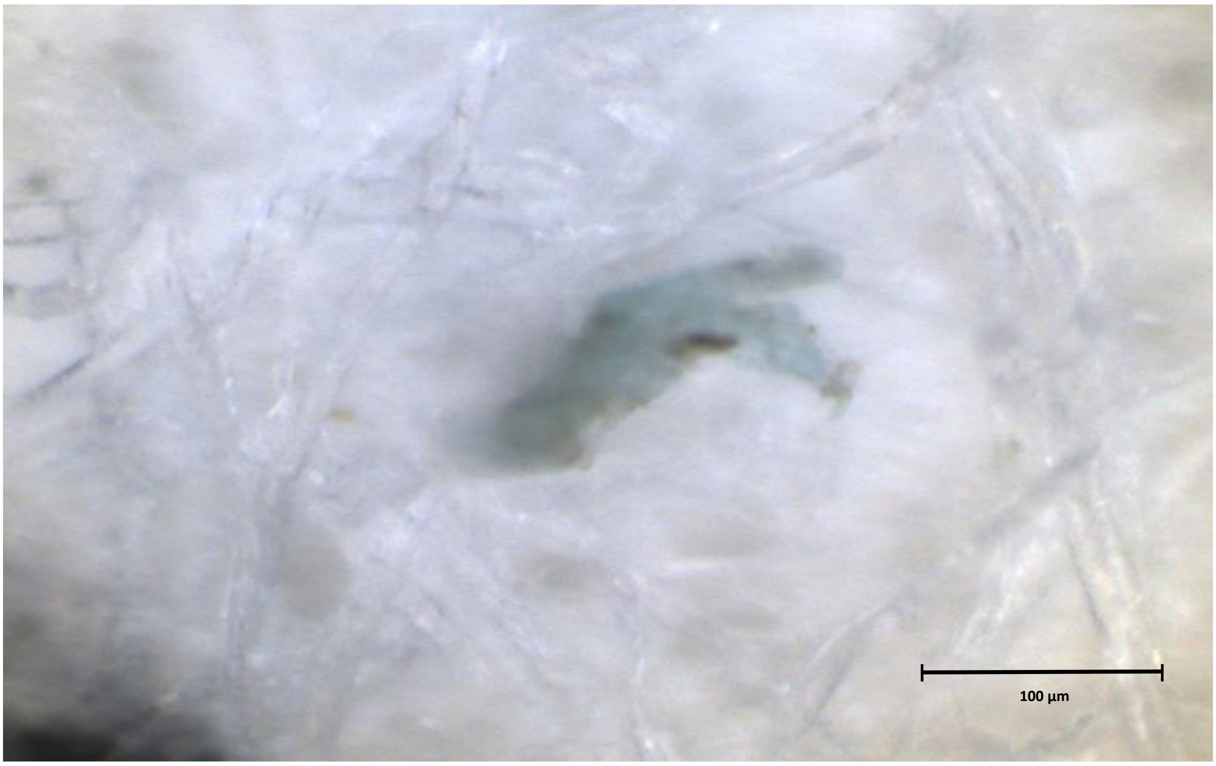

Table S1. Packaging, package weight, origin, salt type, and laboratory controls for 11 salt products purchased in Taipei, Taiwan, in 2017. The types of packaging (columns 2-3) were determined by a combination of information found on the packaging and FTIR spectroscopy (for abbreviations, see Table 1). Columns 7 and 8 give the number of potential microplastic particles for our two types of controls. Column 9 gives the total number of microplastic particles determined by FTIR spectroscopy for the two controls (columns 7 and 8).

Salt ID	Container	Spoon	Weight per package (g)	Origin	Salt type	Control water	Control air	Total
1	Plastic bag (PE)	-	1000	Tainan, Taiwan	Sea salt (refined)	1	-	0
2	Plastic bag (PE)	-	1000	Tainan, Taiwan	Sea salt (refined)	1	-	0
3	Plastic bag (PP)	-	600	Tainan, Taiwan	Sea salt (refined)	0	10	0
4	Plastic bag (PET)	-	400	Taiwan	unidentified	6	11	0
5	Plastic bag (nylon)	-	300	Okinawa, Japan	Sea salt (sun dried)	5	25	0
6	Plastic bag (PET)	-	450	Australia	Sea salt (sun dried)	0	3	0
7	Container (PET), cover lid (PE)	-	480	Italy	Sea salt (sun dried)	2	33	0
8	Box (cardboard)	-	~ 736	USA	Sea salt (sun dried)	2	7	0
9	Container (metal), cover lid (PE)	blue PS	300	Australia and Hualien, Taiwan	Sea salt (sun dried)	2	4	0
10	Container (metal), cover lid (PE)	red PS	300	New Zealand	Sea salt (refined)	0	4	0
11	Plastic bag (PET)	-	1000	Thailand	Mountain rock salt	3	2	0
Total	-	-	-	7 countries	2 types	22	99	0

Figure S1. Examples of spectra generated with FTIR spectroscopy for each of the seven polymer types which we identified among the 43 microplastic particles (for abbreviations, see Table 1). It should be noted that the units on the vertical axis are arbitrary units (see, e.g., Figure 6 in Seth and Shriwastav[1]). The spectra in this figure were produced by the measuring program of the FTIR microscope which we used (see Methods).

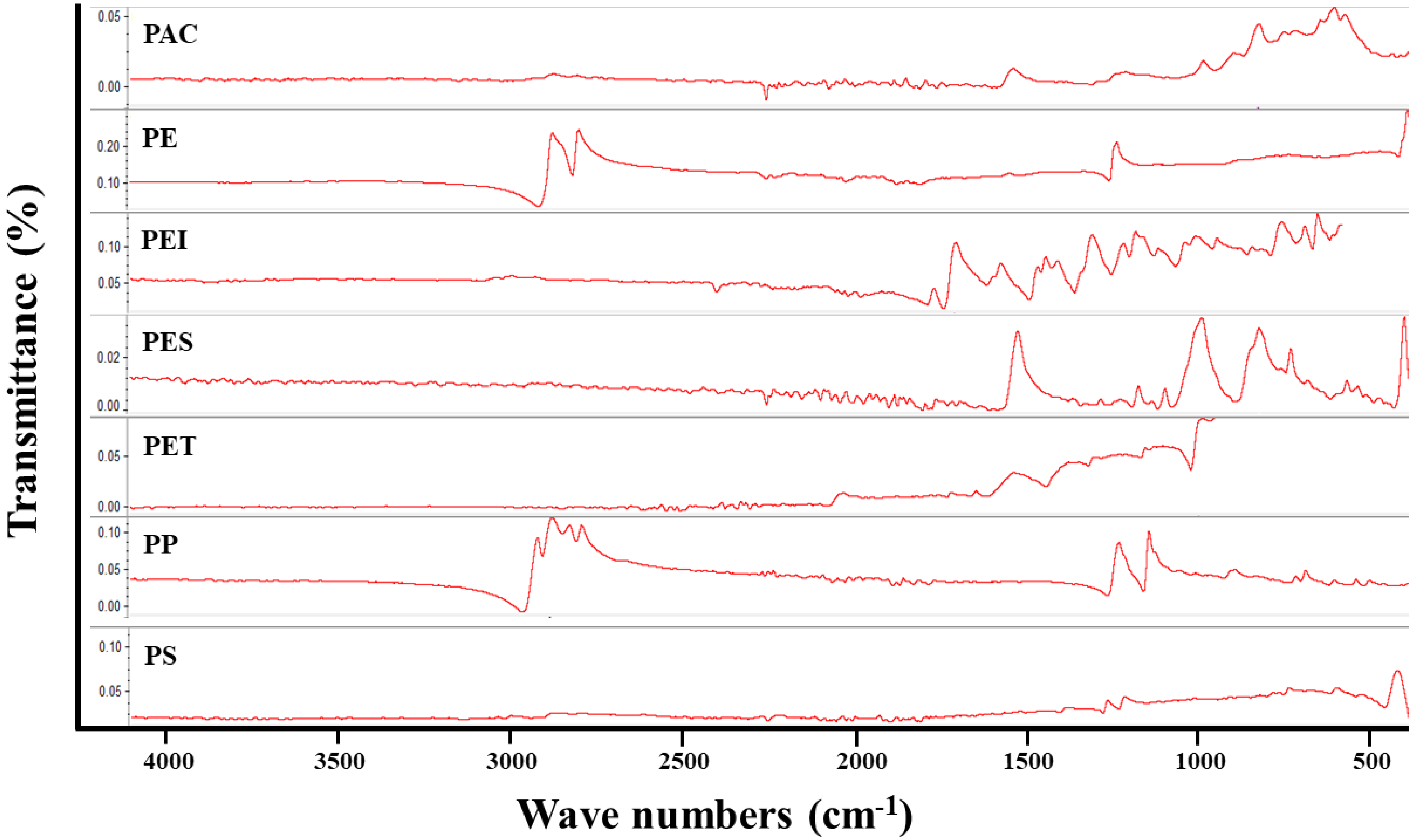

Figure S2. Global geographical distribution of the mean number of microplastic particles/kg of salt. Data were taken from Table 1 for data from our study (orange bars) and taken from Table 2 and references therein for data from other studies (blue bars). The data for Croatia (red asterisk) is not drawn to scale for graphical reasons (the bar for Croatia should be 52 times as long as the bar for Italy, see Renzi and Blašković[2] for details). Data from Karami et al.[3] could not be included because separate data for individual countries are not presented in Karami et al.[3]. Data from Kosuth et al.[4] were excluded because their numbers refer to "anthropogenic particles" (Table 2). Data from Kim et al.[5] were not presented because the data are already depicted in their Figure 2. Note that the large variation depicted in this Figure may be due to "both the variation in the actual contamination of the products ... and the variation in the laboratory procedures" (see Discussion). The map in this figure was produced with the freely available QGIS software (https://qgis.org/en/site/forusers/download.html).

References

1. Seth, C. K. & Shriwastav, A. Contamination of Indian sea salts with microplastics and a potential prevention strategy. *Environ. Sci. Pollut. Res*. **25**, 30122-30131 (2018).
2. Renzi, M. & Blašković, A. Litter & microplastics features in table salts from marine origin: Italian versus Croatian brands. *Mar. Pollut. Bull.* **135,** 62-68 (2018).
3. Karami, A. *et al.* The presence of microplastics in commercial salts from different countries. *Sci. Rep.* **7,** 46173 (2017).
4. Kosuth, M., Mason, S. A. & Wattenberg, E. V. Anthropogenic contamination of tap water, beer, and sea salt. *PLoS ONE* **13,** e0194970 (2018).
5. Kim, J.-S., Lee, H.-J., Kim, S.-K. & Kim, H.-J. Global pattern of microplastics (MPs) in commercial food-grade salts: sea salt as an indicator of seawater MP pollution. *Environ. Sci. Technol.* **52,** 12819-12828 (2018).

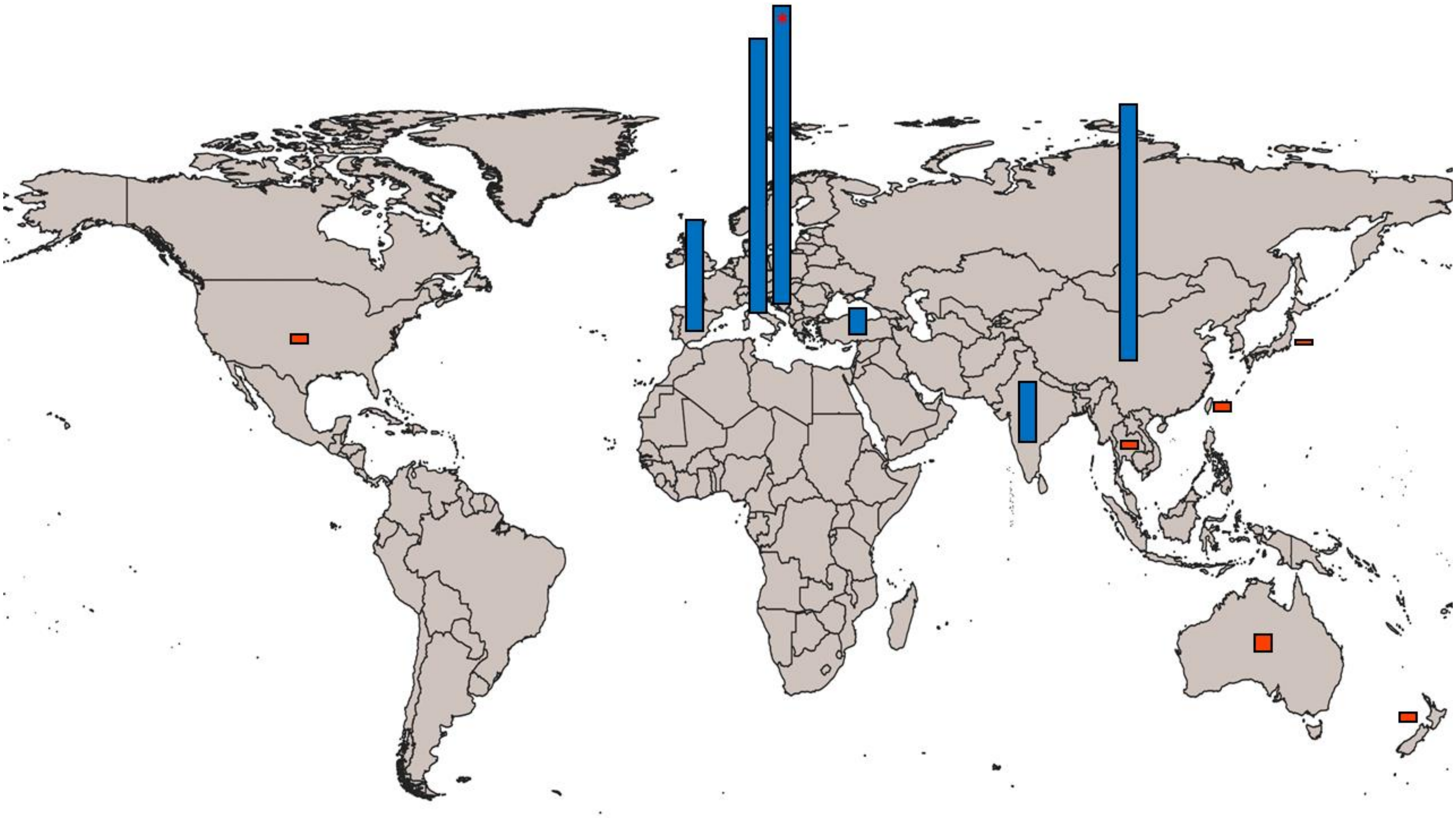

Supplement: Supplementary file 1 — Microplastic contamination of table salts from Taiwan, including a global reviewPlastic pollution is a rapidly worsening environmental problem, especially in oceanic habitats. Environmental pollution [file 41598_2019_46417_MOESM1_ESM.pdf]
